# Microbiome dysbiosis and its modulation in cancer development, prevention and therapy

**DOI:** 10.3389/fonc.2026.1852716

**Published:** 2026-07-02

**Authors:** Shivendra Dixit, Antonia Welker, Diego Ortiz, Marina Athanasouli, Christoph K. Stein-Thoeringer

**Affiliations:** 1 Internal Medicine I, University Clinic Tübingen, University of Tübingen, Tuebingen, Germany; 2 Cluster of Excellence Controlling Microbes to Fight Infection (CMFI), University of Tübingen, Tuebingen, Germany; 3 German Center for Infectious Diseases (DZIF), Partner Site Tübingen, Clinical Research Unit for Microbiome-Host Interactions, Tuebingen, Germany; 4 Genome Biology Unit, European Molecular Biology Laboratory (EMBL), Heidelberg, Germany

**Keywords:** cancer, dysbiosis, microbiome, prevention, therapy

## Abstract

Gut microbiome dysbiosis, a state of microbial imbalance, altered microbial function, and disturbed homeostasis between the gut microbiome and its host, is increasingly recognized as a key contributor to cancer development, progression, and variability in therapeutic response. These microbiome states can facilitate cancer development through chronic inflammation, expansion of microbial genotoxin producers, or disturbances of immune defense mechanisms. In this review, we will discuss current findings on gut microbiome dysbiosis in cancer initiation and progression, emphasizing mechanisms that links dysbiosis to oncogenic transformation and tumor microenvironment remodeling. Furthermore, we will explore microbiome-targeting strategies for cancer prevention and therapeutic support, including dietary modulation, probiotics, prebiotics, and fecal microbiota transplantation. These various microbiome modulations have shown promise in restoring microbial homeostasis, enhancing immunotherapy efficacy, and reducing treatment-associated toxicity. Advances in microbial genomics and metabolomics further enable the identification of biomarkers for predicting cancer risk and therapeutic outcomes. Despite significant progress, translation into clinical settings faces challenges related to interindividual variability, standardization, and mechanistic complexity. Understanding the microbiome-cancer interface provides a platform for personalized, microbiome-informed oncology, paving the way for prevention-driven and precision-guided therapeutics.

## Introduction to microbiome, dysbiosis and cancer

The human microbiome, an intricate ecosystem of trillions of microorganisms predominantly residing in the gastrointestinal tract (1), plays a fundamental role in maintaining host health by regulating nutrient metabolism (2), immune homeostasis (3), and protection against pathogens (4). The constant bidirectional interactions between the host and its commensal microbiome establish a delicate balance essential for a physiological equilibrium. Disruption of this balance, known as dysbiosis, involves shifts in microbial composition and function (5). This can alter microbial metabolic outputs and affect host physiology, contributing to diseases, such as inflammatory bowel disease, metabolic disorders, and cancer (6, 7).

Emerging evidence highlights that specific bacterial species and their metabolites can promote tumor development through various mechanisms including DNA damage, chronic inflammation, and suppression of host immune responses (8). Several mechanistic concepts, such as the “driver-passenger”, “keystone”, and “hit-and-run hypotheses”, have been proposed to describe microbial involvement in tumorigenesis (9–11). In parallel, microbiome-targeting approaches such as dietary interventions, probiotics and fecal microbiota transplantation (FMT) has been explored for cancer prevention or adjuvant treatment strategies. Advances in microbial genomics, metabolomics, and spatial omics have further refined our understanding of microbiome-tumor interactions and facilitated the identification of predictive biomarkers for cancer risk and treatment outcomes.

Despite these advances, significant challenges remain in translating microbiome research into clinical oncology, including high interindividual variability, lack of standardized interventions, and limited understanding of the underlying mechanisms.

This narrative review aims to synthesize current findings on the involvement of dysbiosis in cancer initiation and progression, and its impact on cancer therapy, with emphasis on molecular and cellular mechanisms. In addition to literature search on basic principles of microbiome effects in cancer in general, the scope was to extract evidence for emerging strategies designed to restore microbial homeostasis, including dietary interventions, probiotics, prebiotics, antibiotics, and FMT. Advancing our understanding of the microbiome-cancer interplay may enable the development of personalized, microbiome-informed oncology, providing new opportunities for prevention and precision-guided therapeutics.

## Microbiome and dysbiosis: definitions and mechanisms

### Definition of microbiome dysbiosis

The idea of a healthy microbiome is derived from multiple studies in healthy individuals with no signs of disease. Under healthy conditions, Bacteroidetes and Firmicutes are dominant bacterial phyla in the gut, while Proteobacteria and Actinobacteria are less abundant, but consistently present in most people (12). Dysbiosis can occur across microbiomes at all body sites (i.e., gut, skin, genital, and respiratory tracts), but this review focuses on the gastrointestinal tract.

A stable gut microbiome maintains epithelial integrity, metabolizes dietary substrates into beneficial compounds like short-chain fatty acids (SCFAs), and educates the host immune system toward tolerance and defense (13). Dysbiosis therefore, in simple terms, refers to changes in the composition of the resident microbial community, relative to that found in healthy individuals, and is characterized by a loss of commensals and/or expansion of pathobionts (12). This disrupted state can be triggered by external threats and internal conditions such as malnutrition, exposure to xenobiotics (i.e., antibiotics and other drugs), infections, inflammatory conditions, and lifestyle habits (e.g., alcohol abuse and smoking) (14–16).

Antibiotic treatment disrupts the microbiome by targeting both commensal and pathogenic bacteria. Commonly used antibiotics in clinical care can have significant effects on the microbial composition (17). For instance, vancomycin treatment results in an overall reduction of the diversity of gut commensals, including *Lactobacillus* spp. and *Bifidobacterium* spp., creating ecological niches that favor the expansion of pathogens (18). Broad-spectrum antibiotics, unlike narrow-spectrum agents, exert collateral damage on a substantial fraction of the gut community, causing a rapid decline in richness and evenness, with roughly one-third of resident taxa being affected (19). These antibiotics commonly deplete health-associated members of the Firmicutes, such as *Eubacterium, Ruminococcus, Roseburia*, and *Anaerostipes*, as well as *Bacteroidetes*, many of which are SCFA producers. This loss of diversity and functional redundancy not only weakens colonization resistance against pathogens including *Salmonella enterica, Klebsiella pneumonia* and *Clostridioides difficile*, but also reduces microbial metabolite production, impairing epithelial barrier integrity and host immune homeostasis. Moreover, broad-spectrum antibiotic treatment can result in overgrowth of Proteobacteria such as *Enterobacteriaceae* and opportunistic genera like *Enterococcus* and *Staphylococcus* (20–24).

Dysbiosis and its potential contribution to disease progression has been widely studied in conditions such as inflammatory bowel diseases (IBD), diabetes, and asthma (25–27). These studies have improved our understanding of the processes underlying dysbiosis, which can be broadly categorized into i) loss of beneficial microbes, ii) expansion of pathobionts, and iii) loss of general microbial diversity and microbiome functions (28).

### Dysbiosis, inflammation and host immunity

**Figure 1:**
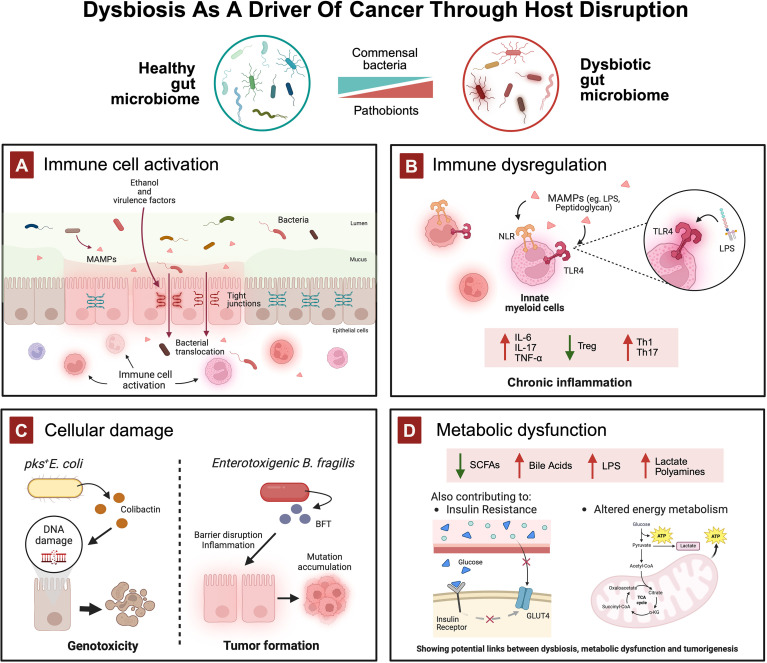
Gut microbiome dysbiosis contributes to cancer development through multiple interconnected host mechanisms. **(A)** Alcohol consumption and virulence factors (e.g. LPS and BFT) compromises intestinal barrier integrity by disrupting epithelial tight junctions, resulting in increased permeability and facilitating translocation of bacteria and microbial products like microbe-associated molecular patterns (MAMPs) like Lipopolysaccharides (LPS) into the lamina propria and systemic circulation. **(B)** Dysbiosis disrupts immune homeostasis by activating innate myeloid cells, including dendritic cells, monocytes, macrophages and neutrophils, through MAMPs. LPS binds to Toll-like receptor 4(TLR4), while other MAMPS like peptidoglycan bind to NOD-like receptors (NLR) on the surface of these cells, promoting pro-inflammatory immune activation and context dependent downregulation of regulatory T cell responses. This results in the production of cytokines such as IL-6, IL-17, and TNF-α, and as a downstream event promotion of Th1- and Th17-type responses, thereby establishing a chronic inflammatory environment. **(C)** Certain microbial strains of *E. coli* and *B. fragilis, in particular pks+ E. coli and enterotoxigenic B. fragilis (ETBF)*, produce genotoxins like colibactin and **(B)** fragilis toxin (BFT) which induce oxidative stress, leading to DNA damage, genomic instability, barrier disruption, inflammation and accumulation of mutations that facilitates tumor initiation and progression. **(D)** Alterations in microbiome composition leads to metabolic dysfunction, characterized by reduced production of beneficial metabolites such as SCFAs and increased levels of potentially harmful metabolites, including secondary bile acids, lactate, and polyamines. In some cases, these changes can promote Insulin resistance and an overall altered energy metabolism and potentially contributing to tumorigenesis.

A healthy gut microbiome promotes immune homeostasis by inducing host anti-inflammatory cytokines like IL-10, thus preventing excessive inflammation. Commensals such as *Bacteroides fragilis* and *Clostridium* spp. stimulate regulatory T (Treg) cells and anti-inflammatory pathways through microbial molecules (e.g., polysaccharide A from *B. fragilis*) binding to host pattern recognition receptors (PRRs) such as Toll-like receptor 2 (TLR2). These mechanisms help restrain inflammation and preserve the mucosal tolerance under healthy conditions (29). However, dysbiosis can significantly alter immune signaling, metabolic outputs, and barrier functions, contributing to a pro-tumorigenic microenvironment (**Figure 1**) (30). During dysbiosis, the loss of beneficial commensals and gut barrier disruption allows translocation of microbial-associated molecular patterns (MAMPs) such as lipopolysaccharide (LPS) across the epithelium (**Figure 1A**). They can trigger innate immune receptors, including TLRs and NOD-like receptors, leading to the activation of NF-κB and STING signaling pathways and sustained production of pro-inflammatory cytokines such as TNF-α, IL-6, and IL-17 (**Figure 1B**) (31). This chronic inflammatory environment drives the recruitment of immunosuppressive cells including myeloid-derived suppressor cells (MDSCs) and Treg cells, thereby suppressing anti-tumor immunity (32, 33).

A notable bacterium that modulates anti-tumor immunity is *Fusobacterium nucleatum (F. nucleatum)*. It is consistently enriched in colorectal cancer (CRC) tissues compared with adjacent healthy mucosa. In microsatellite stable (MSS) tumors, often classified as “cold” tumors, *F. nucleatum* suppresses anti-tumor immune responses by inhibiting the activation and proliferation of NK-cells, CD8+ and CD4+ T cell through virulence factors like FadA and Fap2. FadA activates the E-cadherin/β-catenin pathway, thereby promoting DNA damage, while Fap2, an outer surface protein of *F. nucleatum* binds to the inhibitory receptor TIGIT on NK- and T cells, impairing their cytotoxic activity (34, 35).

In the context of microsatellite instability–high (MSI-H) or deficient mismatch repair (dMMR) tumors, which are typically immunogenic due to high neoantigen load and dense tumor-infiltrating lymphocytes (36, 37), *F. nucleatum* has been implicated in additional tumor-promoting processes. These include the induction of DNA methyltransferase activity and epigenetic remodeling, potentially contributing to the silencing of tumor suppressor genes (37, 38), as well as modulation of epithelial barrier and antimicrobial responses (39).

At first glance, the pro-tumorigenic activity of *F. nucleatum* appears paradoxical in MSI-H tumors, which are considered immunologically “hot,” exhibiting favorable prognosis and strong responses to PD-1 blockade (36, 40). However, this apparent contradiction can be reconciled by considering *F. nucleatum*–driven intra-tumoral heterogeneity. While many MSI-H tumors retain a highly immunogenic phenotype, increasing evidence suggests that tumors with higher intratumoral *F. nucleatum* burden exhibit localized immunosuppressive features. Specifically, *F. nucleatum* abundance has been associated with reduced T-cell infiltration, impaired cytotoxic function, and enrichment of pro-tumorigenic myeloid populations, including M2-like macrophages (38, 41, 42).

Importantly, these microbially influenced niches do not negate the overall immunogenicity of MSI-H tumors, but instead create a state best described as “inflamed yet functionally restrained”. In this context, persistent microbial stimulation may contribute to T-cell dysfunction and exhaustion, potentially mediated through NF-κB signaling and upregulation of immune checkpoints such as PD-L1 (43, 44). This framework helps explain how MSI-H tumors can display favorable cohort-level outcomes and responsiveness to immunotherapy, while still harboring subgroups with relative immune resistance and poorer clinical trajectories.

Crucially, the immunomodulatory capacity of *F. nucleatum* extends well beyond the colorectal niche, establishing it as a broader architect of tumor immune evasion across various malignancies. In gastric cancer, for example, recent evidence demonstrates that intratumoral *F. nucleatum* actively recruits tumor-associated neutrophils (TANs). These TANs are driven toward a pro-tumoral phenotype that upregulates PD-L1, directly inducing CD8^+^ T-cell exhaustion and facilitating immune escape (45). Similarly, in oral squamous cell carcinoma (OSCC) and esophageal squamous cell carcinoma (ESCC), *F. nucleatum* activates signaling cascades such as the STAT3 pathway and the NLRP3 inflammasome that enrich the microenvironment with immunosuppressive myeloid-derived suppressor cells (MDSCs) and pro-inflammatory cytokines like IL-1β (46). Furthermore, owing to its capacity for systemic translocation and its high affinity for the Gal-GalNAc receptor overexpressed on many host tissues, *F. nucleatum* colonization has been increasingly implicated in shaping the metastatic and immunosuppressive landscapes of extra gastrointestinal malignancies, including breast and pancreatic tumors (46, 47).

Taken together, these observations support a model in which *F. nucleatum* acts as a context-dependent modifier of the tumor immune microenvironment. By contributing to intra-subtype heterogeneity and driving localized immune evasion, it not only influences therapeutic responses in MSI-H CRC, but also increasingly emerges as a pan-cancer driver of carcinogenesis, metastasis, and global immunoresistance.

### Dysbiosis and intestinal barrier damage

Under homeostatic conditions, host cells and commensal microbes contact is limited by mucus and antimicrobial peptide (AMP) layers, acting like physical barriers that prevent excessive immune activation (48). Dysbiosis disrupts key mechanisms that preserve intestinal barrier integrity, including AMP secretion, epithelial renewal, and tight junction organization, increasing permeability and inducing inflammation (**Figure 1A**) (49).

A central component of this regulation is AMP production by Paneth cells, particularly human α-defensins 5 and 6 (HD5 and HD6), which help shape the composition of the intestinal microbial community (50, 51). Perturbations in Paneth cell function or defensin expression favor expansion of pathobionts, subsequent barrier failure, and chronic mucosal inflammation (52). In parallel, circadian clocks in intestinal epithelial cells coordinate daily oscillations of barrier-relevant proteins, such as tight junction proteins and mucins, synchronizing the barrier function with fluctuations in microbial and dietary cues. Disruption of these circadian rhythms has been associated with changes in the microbiome composition, increased intestinal permeability, and increased susceptibility to inflammatory and neoplastic processes (53).

Dysbiosis can also enable specific bacteria to exert direct, structurally damaging effects on the epithelial barrier. Enterotoxigenic *B. fragilis* (ETBF) is a well-characterized example; its toxin cleaves E-cadherin, a junctional adhesion protein, thereby compromising epithelial cohesion, increasing paracellular flux, and activating pro-inflammatory signaling pathways (54, 55). Similarly, certain *Enterococcus* strains impair tight junction architecture by producing enzymes such as gelatinase (GelE), facilitating translocation of luminal antigens and microbes, which in turn amplifies mucosal immune activation and sustains inflammation (56, 57) (**Figure 1C**). Collectively, these mechanisms illustrate how altered microbial communities and their products converge on the intestinal barrier to drive a state of persistent inflammation that can favor tumor-promoting conditions in the gut.

### Microbial toxins and DNA damage of host cells

**Figure 2:**
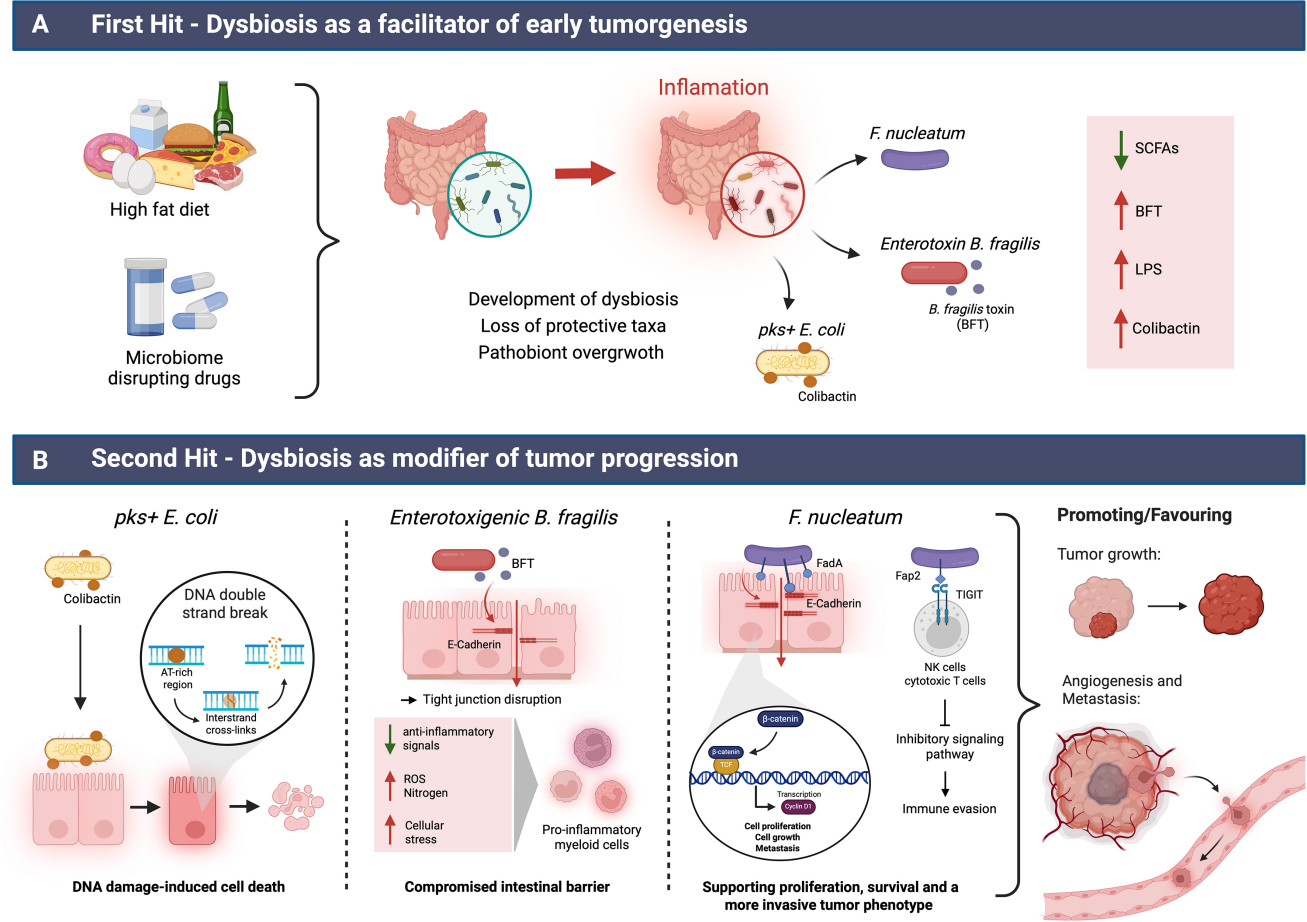
Two-step contribution of dysbiosis to carcinogenesis. **(A)** First Hit: Loss of protective gut taxa through high fat diets and use of microbiome disrupting drugs(e.g. antibiotics) leads to a development of dysbiosis and expansion of pathobionts such as *F. nucleatum*, *pks^+^ E. coli* and *enterotoxigenic B. fragilis.* This shifts the microbial ecosystem toward increased levels of Lipopolysaccharides (LPS), *B. fragilis toxin* (BFT), and colibactin, with concomitant depletion of short-chain fatty acids (SCFAs), which induces inflammation by activation of the immune system. **(B)** Second Hit: Concurrently, dysbiosis driven colonization by *pks+ E. coli* at the mucosal surface introduces the genotoxin colibactin, which forms interstrand crosslinks at AT-rich motifs in host DNA that are subsequently resolved into double-strand breaks. The resulting genotoxic stress, compounded by sustained myeloid cell inflammation and microbial immune evasion, drives persistent epithelial DNA damage and cell death, that accelerates the transition from precancerous lesions to invasive carcinoma. *B. fragilis* toxin (BFT) disrupts the intestinal epithelial barrier by cleaving E-cadherin and as a downstream event destabilizes tight junction components such as occludin and claudins, leading to a compromised intestinal barrier and therefore allowing luminal contents including LPS and bacteria to translocate across the epithelium. *F. nucleatum* adhere via its surface protein FadA to epithelial cells and cleave E-cadherin, which as well leads to disruption of epithelial cells. This triggers a downstream event of β-catenin induced transcription factor activation and production of cyclin D1, which contributes to enhanced cell growth and proliferation. Binding of *F. nucleatum* via Fap2 to the T cell immunoreceptor with Ig and ITIM domain (TIGIT) leads to a downregulation of NK cells and cytotoxic t cells and therefore to an immune evasion. All together these events fuels tumor growth, angiogenesis and promotes metastatic dissemination.

Bacterial genotoxins facilitate mutagenesis in epithelial cells and DNA damage through alkylation, oxidative stress induction, or cleavage of DNA strands (**Figure 1C**) (54, 58, 59). For instance, *pks^+^ Escherichia coli* carries a 50 kb hybrid polyketide-non-ribosomal peptide synthase operon (*pks*), which is responsible for the production of the genotoxin colibactin (60). *pks*^+^ *E. coli* causes interstrand crosslinks and double strand breaks in epithelial cell lines and in mouse models of CRC. Colibactin alkylates DNA leading to single base substitution (SBS) and small insertion-deletion (indel) signatures, characterized by single thymine deletions (**Figure 2B**) (61). This bacterial genotoxin has become a potential biomarker for risk stratification, and a target with interventions focusing on its biosynthetic pathways and key involved genes to mitigate microbiota-driven tumorigenesis. For example, the use of boronic acid-based inhibitors to target colibactin-activating peptidase (ClbP) has been explored (62). However, such strategies are still experimental and require further investigation before clinical application. High levels of *pks*^+^ *E. coli* and ETBF have been identified in tumor tissues and fecal samples of CRC patients. Toxin-derived damage activates host DNA repair mechanisms, but chronic exposure can overwhelm repair capacity, leading to mutation accumulation. Over time, this promotes oncogene activation and tumor suppressor gene inactivation, contributing to the initiation and progression of CRC and other gastrointestinal cancers (63).

### Dysregulation of the metabolic function of the microbiome in dysbiosis

Gut microbiome dysbiosis can worsen host glucose and fat metabolism, promoting obesity, fatty liver disease, and diabetes, and these metabolic changes together with microbial signals create a pro−cancer environment in several organs (64–66). In obesity, shifts toward energy-harvesting microbial consortia enhance dietary calorie extraction and fat deposition while reducing anti-inflammatory SCFA production. This fosters systemic low-grade inflammation and insulin resistance, which drive epithelial cell proliferation via hyperinsulinemia and phosphoinositide 3-kinase (PI3K)/Akt signaling (**Figure 1D**) (67–69). Concurrently, diet-induced barrier dysfunction facilitates circulating LPS derived from Gram negative bacteria such as *E. coli* and *K. pneumonia*, promoting metabolic endotoxemia that exacerbates steatosis in metabolic dysfunction-associated steatotic liver disease (MASLD). In this context, dysregulated bile acid pools, choline deficiency, and bacteria such as *Ruminococcus gnavus* further exacerbate lipotoxicity, oxidative stress, and fibrogenesis contributing to progression toward hepatocellular carcinoma (HCC) (64, 70).

*Enterococcus faecalis* (*E. faecalis*) may promote hepatocellular carcinoma development by GelE-mediated intestinal barrier disruption, and subsequent translocation from a dysbiotic gut to the liver. There, it can persist intracellularly within hepatocytes and produce cytolysin, leading to hepatocyte injury and inflammation (71–73). In alcohol−associated liver disease, an important precursor to HCC, ethanol−induced barrier dysfunction and reduced expression of the antimicrobial lectin REG3 favor mucosal overgrowth and translocation of *E. faecalis* to the liver (74, 75). This is particularly relevant for cytolysin−positive strains, which are associated with more severe alcoholic hepatitis and markedly increased short-term mortality due to liver failure (76). Although the precise mechanisms of *E. faecalis* - driven carcinogenesis in HCC remain under investigation one can propose the following hypotheses: (i) chronic inflammation from cytolysin-induced hepatocyte lysis, releasing DAMPs/cytokines (e.g., IL-1β, CXCL8) that recruit immunosuppressive neutrophils/M2 macrophages and sustain fibrosis; (ii) direct genotoxicity via cytolysin pores/DNA damage or GelE proteolysis of tumor suppressors; and (iii) hepatocyte metabolic reprogramming, e.g., *E. faecalis* - delivered Obg GTPase mimicking Rheb to hyperactivate mTOR, boosting proliferation often synergizing in ALD/HCC models (71, 73, 77–79).

These interconnected pathways encompassing systemic hyperinsulinemia, gut-liver inflammatory crosstalk, and local genotoxin exposure collectively frame metabolic dysregulation as a microbiome-mediated gateway that drives carcinogenesis across diverse tissues, including colon, liver, and prostate (80).

## Dysbiosis and cancer development and progression

The role of dysbiosis in cancer pathogenesis can be explained by the “two-hits hypothesis”. In this model, microbiome dysbiosis acts in two distinct, but interconnected phases that contribute to both the initiation and progression of solid cancers like colorectal, gastric, esophageal, hepatocellular cancers, and cholangiocarcinoma (81).

The “first hit” refers to gut microbiome dysbiosis, characterized by a qualitative or quantitative shift favoring the expansion of pathobionts, alongside a loss of beneficial taxa. This dysbiotic state is considered an initiating event in early tumorigenesis and has been observed in adenomas before malignant transformation. Although dysbiosis alone increases susceptibility, additional events (“second hit”) such as genotoxin exposure, inflammation and immunosuppression are typically required to drive tumorigenic changes (**Figure 2A**) (81, 82).

As an example, increasing evidence implicates gut dysbiosis in the earliest stages of pancreatic ductal adenocarcinoma (PDAC) development, well before overt carcinoma is established (83, 84). Dysbiosis compromises intestinal barrier integrity, elevates systemic levels of microbial products such as LPS, and reshapes metabolite profiles, collectively inducing chronic low−grade systemic inflammation and immune dysregulation within the pancreas (85, 86). These circulating mediators reach the pancreas, where they activate pattern−recognition receptors on acinar, ductal and immune cells, driving acinar stress responses, acinar−to−ductal metaplasia (ADM) and an immunosuppressive microenvironment (87). In preclinical mouse models, dysbiotic conditions are associated with expansion of MDSCs and Treg cells, coupled with impaired cytotoxic T−cell activity around early pancreatic intraepithelial neoplasia (PanIN) lesions (85, 86). In this model, a “first hit” gut dysbiosis does not accelerate the progression of established PanINs, but rather creates a PanIN−permissive milieu: Acinar/ductal cells in a histologically normal pancreas are subjected to persistent inflammatory and metabolic stress, increasing their susceptibility to oncogenic mutations and reducing the likelihood that the earliest transformed clones will be cleared by immune surveillance (88, 89).

In individuals with colorectal adenomas, microbial alterations are detectable despite histologically normal mucosa. The microbial communities in these cases are already predominantly Proteobacteria and potential pathogens (e.g., *Pseudomonas, Fusobacterium, toxin producing Bacteroides* spp.), with a concomitant reduction in beneficial metabolite producing commensals (90). For instance, the expansion of pathogenic effects of ETBF (91) may result in enhanced epithelial proliferation, a Th17 cell−skewed inflammatory milieu and increased production of reactive oxygen (ROS) and nitrogen species, thereby imposing chronic genotoxic and proliferative stress on an otherwise healthy mucosa (92, 93). The concomitant loss of SCFA−producing Firmicutes further diminishes anti−inflammatory and pro−apoptotic signals in the intestinal milieu, shifting colonocyte metabolism from butyrate oxidation toward alternative substrates that favor proliferation over differentiation (**Figure 2A**) (94, 95).

The “second hit” refers to downstream consequences of dysbiosis, which include sustained low-grade inflammation, genotoxicity, impaired anti-tumor immunity and cellular stress responses. Collectively, these factors accelerate the progression from precancerous states to invasive neoplasia and metastasis by reshaping the tumor microenvironment and evading immune clearance (81). For instance, metagenomic analyses of fecal samples from CRC and adenoma patients have identified an enrichment of pathobionts, including *pks^+^ E. coli*. These bacteria produce genotoxins, such as colibactin, which induce DSBs and characteristic mutational signatures, representing a potential “second hit” in cancer development (**Figure 2B**) (96).

This model underscores that dysbiosis is not merely a transient event, but a persistent and dynamic factor in cancer biology. Acting as both an initiator and ongoing modifier of disease, the microbiome represents a continuously relevant target not only for cancer prevention but also for improving treatment responses and limiting disease progression.

## Dysbiosis and cancer therapy

Beyond its role in cancer development and progression, dysbiosis influences the efficacy and toxicity of cancer treatment across multiple therapeutic modalities. The gut microbiome can either enhance or impair treatment responses, by modulating drug metabolism, immune system priming, and the baseline inflammatory state of the host. This bidirectional relationship between microbial community compositions and therapeutic outcomes has been documented across immunotherapy, chemotherapy, as well as radiotherapy, and is increasingly positioning the microbiome as a clinically relevant target for intervention.

### Dysbiosis and immunotherapy

Cancer immunotherapy has been fundamentally transformed since immune checkpoint inhibitors (ICI) and chimeric antigen receptor (CAR) T cell therapies are available. Their efficacy, however, is not determined by the drug alone. Evidence from human studies and clinical trials showed that the gut microbiome emerges as a decisive co-factor influencing response, resistance, and toxicity in these therapies (97, 98).

In ICI, gut dysbiosis can compromise positive therapy outcomes by depleting metabolically active bacteria such as Firmicutes, Actinobacteria and especially *Faecalibacterium prausnitzii*, which SCFA production, tryptophan-derived indoles, and secondary bile acids, that together regulate T-cell differentiation, gut barrier integrity, and systemic immunity (99, 100). In addition, SCFAs enhance the effector functions of anti-tumor CD8^+^ T cells by inhibiting histone deacetylases and activating the mechanistic target of rapamycin (mTOR), while promoting Treg cell differentiation through GPR109A signaling pathways on dendritic cells. Without adequate SCFA levels, CD8^+^ T cell effector function and metabolic fitness decline, which leads to a compromised ICI efficacy (101). Clinical and preclinical data consistently show that a favorable ICI response is associated with enrichment of specific taxa as mentioned above. Conversely, antibiotic exposure prior to ICI treatment is associated with worse clinical outcomes and blooms of unfavorable species. The microbiome influences drug metabolism and immune activation, while ICI and co-administered chemotherapy in turn can reshape microbial communities, highlighting a bidirectional relationship (102, 103).

Patients undergoing cellular cancer therapies often require antibiotic treatment, because lymphodepleting chemotherapy severely compromises the immune system, creating a window during which patients are highly vulnerable to bacterial infections. Use of broad-spectrum antibiotics (e.g., piperacillin-tazobactam and meropenem) is therefore clinically necessary to treat life-threatening infections. However, this leads to a severe reduction of gut microbial diversity at expense of *Roseburia*, *Bifidobacterium* or *Ruminococcus* spp., exacerbating dysbiosis and enriching pathobionts such as *Enterococcus, Streptococcus or Klebsiella* spp. with pro-inflammatory properties (104). Consequently, LPS released by these pathobionts binds to Toll-like receptor 4 (TLR4) on myeloid cells, amplifying NF-κB activation and IL-6/IL-1β storms, which may in turn suppress the antitumor efficacy of cell therapeutics, e.g., CAR T cells (105). This tension between infection prevention and microbiome preservation represents an active area of clinical investigation, with emerging interest in whether selective antibiotic strategies or microbiome restoration approaches via FMT could mitigate these effects (106).

### Dysbiosis and chemotherapy

In recent years several associations between the gut microbiome and chemotherapy response and toxicity have been found in preclinical and clinical studies. For instance, the gut microbiome has an impact on chemotherapy by directly modifying or metabolizing anticancer drugs, leading to a higher toxicity and bioaccumulation of the chemotherapeutics that have been used in the treatment (107). For example, *Bacteroides* spp., *F. prausnitzii* and *Clostridium* spp., are β-glucuronidase producers, that can convert the prodrug irinotecan (CPT-11) into its active form (SN-38) in the gut, where it is accumulated and leads to diarrhea (107). In contrast, intratumor Gammaproteobacteria expressing a long isoform of cytidine deaminase (CDD_L_) metabolize gemcitabine into its inactive form, thereby conferring tumor resistance to this chemotherapeutic agent. This has been demonstrated in colon cancer models and PDAC tumoral tissue, where 76% of tumors harbored such bacteria. Antibiotic cotreatment (e.g., ciprofloxacin) abrogated resistance by eliminating the bacteria, highlighting a potential therapeutic strategy to enhance chemotherapy efficacy (108, 109).

Gut bacteria have been proven to metabolize orally administered chemotherapeutics through pathways shared with human hosts. For instance, many commensal bacteria harbor the *preTA* operon, which converts fluorouracil (5-FU) into its inactive metabolite dihydrofluorouracil. These findings suggest that inter-individual microbiome differences may partly explain variability in drug response (110). Other studies have shown that bacterial species can modulate chemotherapy activity in both positive and negative directions. A systematic screen of 30 chemotherapeutic drugs demonstrated that bacteria such as *E. coli* and *Listeria welshimeri* can either reduce or enhance drug cytotoxicity depending on the compound. For example, cladribine and daunorubicin activity was reduced, whereas drugs like fludarabine and CB1954 became more cytotoxic in the presence of bacteria (111).

The gut microbiome can also indirectly modulate chemotherapy through systemic metabolic pathways. For example, microbial metabolism of dietary phytochemicals induces hepatic cytochrome P450 enzymes, accelerating the clearance of PI3K inhibitors and reducing their anticancer activity in mice. Diets lacking these phytochemicals or transient antibiotic treatment restored drug efficacy, revealing a microbiome-diet-liver axis that acts as a function modified of therapy response (112).

Chemotherapy toxicity is also influenced by the microbiome, illustrating a bidirectional relationship. Reduction of mucus degrading bacteria in the gut microbiome has been implicated in the development of mucositis, a frequent adverse event in 5-FU chemotherapy (113, 114). Furthermore, chemotherapeutic drugs can also directly perturb the gut microbiome. A large-scale *in vitro* drug screen revealed that antimetabolites, antipsychotics and calcium-channel blockers inhibit commensal bacteria more than other medications (115). Fecal samples collected after chemotherapy in non-Hodgkin’s lymphoma patients revealed a significant decrease in beneficial bacteria like Firmicutes and Actinobacteria, alongside a significant increase in Proteobacteria (116). In mouse models, interventions such as prebiotic supplementation or FMT reduced chemotherapy-induced side effects, including mucositis and dysbiosis in the gut, illustrating potential strategies to mitigate adverse effects and improve treatment outcomes (117).

### Dysbiosis and radiotherapy

Radiotherapy (RT) is a central treatment modality for pelvic malignancies. However, its interaction with the microbiome remains poorly understood. Pelvic RT may promote dysbiosis through direct epithelial damage and oxidative stress, leading to reduced microbial α-diversity and an expansion of Proteobacteria (118, 119). *In vitro* studies using microbiota derived from patients with radiation enteritis have demonstrated induction of epithelial inflammation and barrier dysfunction in bacterial-epithelial co-culture systems, accompanied by increased TNF-a and IL-1B expression compared to control microbiota (120).

Concurrently, radiation-induced epithelial damage has been associated with disruption of tight junction integrity, via decreased expression of claudin-1, occludin and ZO-1 and thereby promoting microbial translocation (121). The combined effects of microbiome alterations and epithelial barrier disruption contribute to the development of radiation enteritis, a condition affecting up to 80% of pelvic radiotherapy patients and representing a major dose-limiting toxicity (122). In this context, dysbiosis is not merely a consequence of tissue injury, but also an active amplifier of mucosal inflammation, creating a feed-forward loop that exacerbates gastrointestinal toxicity.

In contrast, specific microbial configurations appear to confer radioprotective effects. A Firmicutes-dominant microbiome enriched in short-chain fatty acid (SCFA)-producing taxa, particularly butyrate producers, has been associated with improved epithelial barrier integrity and reduced inflammatory injury. Butyrate, in particular, promotes epithelial repair, enhances tight junction assembly, and exerts anti-inflammatory effects on mucosal immune cells, suggesting a mechanistic basis for microbiome-mediated protection against RT-induced damage (123–126).

Beyond local toxicity, emerging evidence indicates that the microbiome also modulates systemic anti-tumor immunity induced by RT, including the rare but clinically significant abscopal effect (127). However, the immunomodulatory properties of SCFAs like butyrate act as a double-edged sword. While their anti-inflammatory effects protect healthy mucosal tissue locally, their systemic elevation can paradoxically impair anti-tumor immunity. This phenomenon is mediated by radiation-induced activation of the cGAS - STING pathway, type I interferon (IFN-β) signaling, and CD8^+^ T-cell priming, leading to regression of distant, non-irradiated tumors (128, 129). Preclinical studies suggest that microbial composition heavily influences the magnitude of these systemic immune responses, though the dynamics differ significantly from pure immunotherapy. Interestingly, while antibiotic-induced dysbiosis generally dampens responses to systemic immunotherapy, its role in radiotherapy is highly context-dependent. Preclinical models have shown that certain antibiotics, such as vancomycin, can paradoxically *enhance* the RT-induced abscopal effect by depleting specific immune-suppressive bacterial populations. Furthermore, while systemic SCFAs like butyrate protect the local gut epithelium from radiation toxicity, elevated systemic levels have been shown in some studies to impair cross-priming and abrogate radiation-induced systemic anti-tumor immunity (130, 131).

Collectively, these findings highlight a dual role of the gut microbiome in radiotherapy: (i) modulation of treatment-related gastrointestinal toxicity through maintenance of epithelial barrier integrity, and (ii) regulation of systemic anti-tumor immune responses that underpin the abscopal effect. This positions the microbiome as a potential therapeutic target, with dietary interventions, probiotics, and microbiota-based therapies emerging as promising strategies to mitigate toxicity while enhancing immunogenicity of radiotherapy (132, 133).

## Preventing and treating dysbiosis: microbiome modulations for cancer prevention and therapy

### Dietary interventions and prebiotics

Dietary imbalance plays a critical role in shaping cancer onset, progression, and susceptibility to treatment- or disease-related complications (134). A large-scale meta-analysis encompassing 860 comparisons systematically assessed how various dietary elements relate to cancer risk across different organs and identified numerous significant correlations (135). Another recent study including 1.8 million participants across the UK, US, Taiwan and India with a median follow-up of 16 years, also studied the effects of dietary habits on cancer risks, and identified specific dietary patterns associated with specific cancer risks (136). Western-style diets, characterized by their high-fat, low-fiber, high-refined sugar and ultra-processed foods are among the leading suspects in the pathogenesis of a wide range of cancers including pancreatic, colorectal, ovarian, breast, and prostate cancers, as well as CRC metastasis (137, 138). High-fat diets are usually accompanied by low intake of non-digestible fermentable carbohydrates (NDFCs), impairing butyrate production by gut bacteria, promoting colitis. Reduced butyrate levels also affect colonocyte bioenergetics and increase oxygen in the lumen, facilitating the expansion of facultative anaerobes implicated in colonic carcinogenesis, such as *pks*^+^ *E. coli*, non-typhoidal *Salmonella*, *Morganella morganii*, *Actinomyces odontolyticus* and *Campylobacter jejuni* (134, 139, 140).

In contrast, a high-fiber diet consisting of vegetables, whole fruits, whole grains, nuts and legumes, omega-3 fatty acids, and polyunsaturated fat has been associated with lower overall mortality in multiple cancer types. Diets with low glycemic levels are metabolized and absorbed slowly, which results in a gradual increase of insulin levels, associated with reduced risk of CRC and other types of cancer (141–143). These diets are associated with higher abundances of beneficial metabolite producing *Bifidobacterium*, *Lactobacillus, Oscillospira*, and *Roseburia* spp., and a reduction of proinflammatory markers (144–146). However, while these associations hold consistently in colorectal cancer cohorts, evidence from pancreatic and hepatocellular carcinoma studies is more mixed, with smaller effect sizes likely due to differences in microbial exposure routes via the gut-liver axis and lower baseline fiber fermentation capacity (147).

Diet components can shape the microbiome through several mechanisms; micronutrients may selectively stimulate the proliferation of certain taxa while suppressing others. An example of this is seen in diets rich in processed meats containing dietary nitrates which are converted into nitrites, precursors of carcinogenic nitrosamines by species such as *Veillonella* and *Actinomyces* (148, 149). Nutrient imbalance can also compromise intestinal barrier integrity and indirectly alter microbial ecology. An example of this is vitamin D deficiency which weakens gut barrier function, allowing the expansion of potentially opportunistic pathogenic commensals while suppressing beneficial species (150).

**Figure 3:**
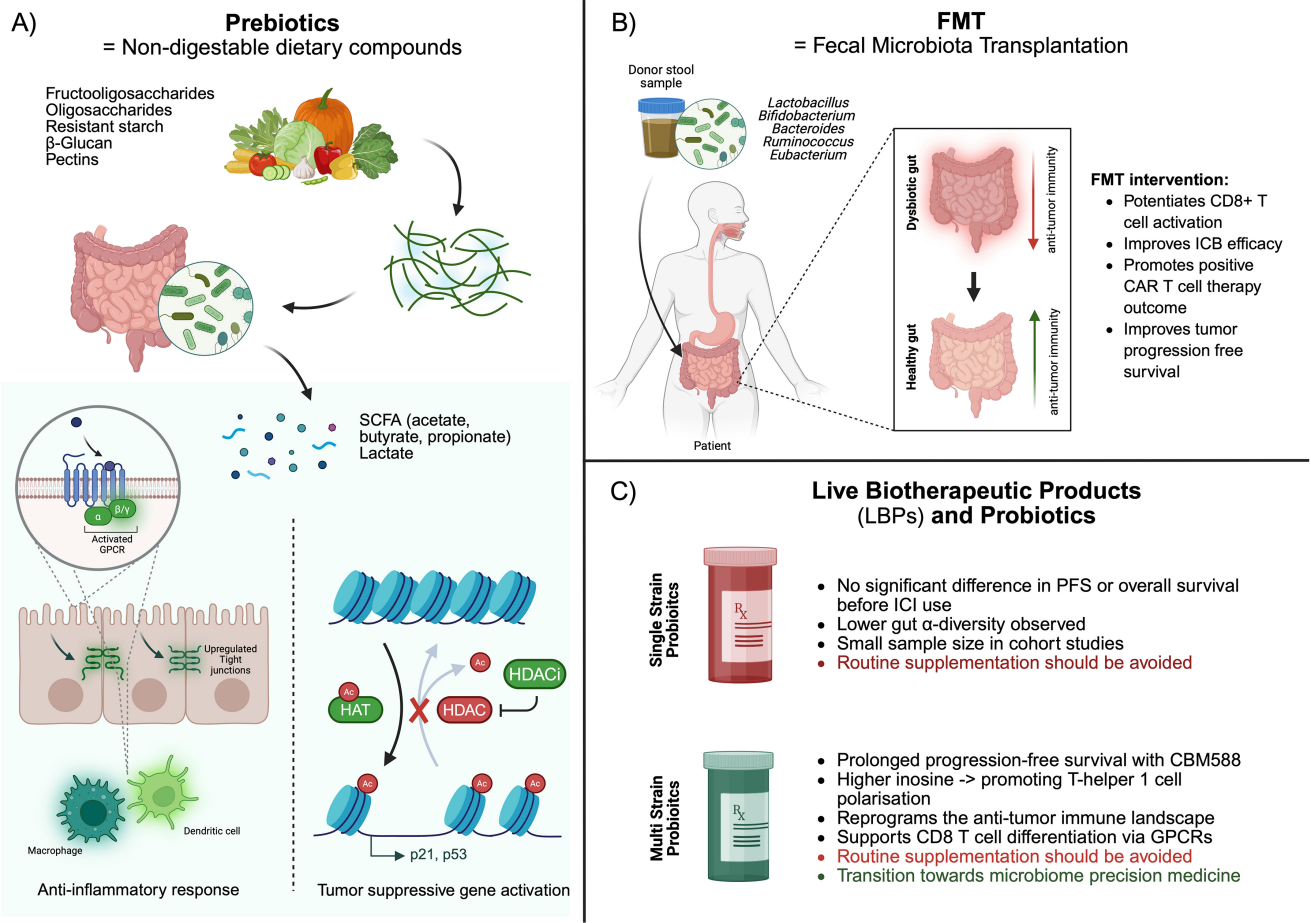
Microbiome-targeted therapeutic strategies and their impact on host physiology and cancer outcomes. **(A)** Prebiotics, defined as non-digestible dietary components such as oligosaccharides, resistant starch, β-glucans, and pectin´s, selectively promote the growth and activity of beneficial gut microbiota. Their fermentation leads to the production of microbial metabolites with several effects on the host, like upregulation of tight junctions in the intestinal barrier through GPCR signaling and transcription of tumor suppressive genes (p21 and p53) via inhibition of the histone deacetylase (HDAC) enzyme. **(B)** FMT involves the transfer of microbial communities from a healthy donor to a recipient with dysbiosis, aiming to restore microbial diversity and composition. FMT has been associated with improved anti-tumor immune responses and improved clinical outcomes. These effects are potentially linked to the re-establishment of a more balanced gut microbial ecosystem. **(C)** LPBs and probiotics, including single- and multi-strain formulations, are being explored for their role in modulating the gut microbiome and host immunity. While single-strain probiotics have shown limited and variable clinical benefit, multi-strain formulations may influence antitumor immunity.

Prebiotics are non-digestible dietary components that are instead fermented by selected gut microbes, thereby linking diet and microbiome, and playing a role in cancer-relevant host responses (151, 152). These include specific fibers and oligosaccharides such as inulin-type fructans, fructooligosaccharides, galactooligosaccharides, resistant starch, β-glucans, and pectins, which are abundant in plant foods like chicory, onions, garlic, leeks, whole grains, and legumes (153, 154). By serving as substrates for beneficial taxa such as *Bifidobacterium, Lactobacillus, Roseburia*, and *Faecalibacterium*, prebiotics enhance acetate, propionate, and butyrate production supporting colonocyte energy metabolism and mucosal immunity (155, 156). Consistent with this, prebiotics can promote the expansion of Treg cells and modulate dendritic cells, neutrophils, and monocytes, shifting immune responses away from chronic, tumor-promoting inflammation towards anti-tumor immunity (**Figure 3A**) (157). Prebiotic efficacy appears most robust in preclinical models of colitis-associated CRC, but shows more variable immune modulation in established tumors, likely reflecting differences between prevention and treatment contexts.

### Fecal microbiota transplantation

FMT has emerged as a promising approach to enhance responses to cancer immunotherapies by modulating gut microbiome composition and function (106). Clinical observations in melanoma patients revealed that transferring fecal material from patients who respond to ICI therapy into non-responders can induce clinical benefit in a subset of cases, indicating a functional role of commensal microbes in shaping antitumor immunity (**Table 1**; NCT03341143, **Figure 3B**) (106). Mechanistic studies suggest that specific taxa and their metabolites, such as *Lactobacillus reuteri* derived indole-3-aldehyde and *Bifidobacterium pseudolongum* derived inosine, can potentiate CD8^+^ T cell activation and improve ICI efficacy (180). The randomized phase II TACITO trial (NCT04758507) in renal cell carcinoma showed improved progression-free survival with FMT plus ICIs, accompanied by donor strain engraftment and correction of antibiotic-induced dysbiosis (**Table 1**) (159). Another recently published phase I PERFORM trial (NCT04163289) showed that healthy donor FMT improved ICI efficacy, while reducing immune-related adverse events (166). Extending this approach directly to gastrointestinal malignancies, the recent phase II RENMIN-215 trial (ChiCTR2100046768) demonstrated the safety and promising clinical efficacy of combining FMT with tislelizumab and fruquintinib in patients with refractory microsatellite stable (MSS) metastatic colorectal cancer, successfully challenging the historical immune resistance of these tumors (167). However, FMT faces substantial challenges, such as donor selection and screening, manufacturing logistics, viability of anaerobes, cold chain requirements, regulatory oversight, and unknown long-term safety (181, 182).

**Table 1:** Summary of clinical trials related to microbiome intervention (FMT, LBPs, and Probiotics) in various cancer types.

Study/ID	Condition	Intervention & regimen	N	Microbiome changes	Primary clinical endpoint	References
NCT04130763	Gastrointestinal Tract Cancer	FMT, Healthy donors via Oral Capsules + Anti-PD-1	10	Responders (R): ↑ α-diversity, ↑ donor-like β-diversity convergence, & ↑ colonization with strain-level convergence.	Objective Response Rate (ORR)	(158)
NCT04758507 (TACITO)	Renal Cell Carcinoma	FMT, ICI responders via Colonoscopy + Capsules + Prembrolizumab + Axitinib	50	d-FMT vs. p-FMT: ↑ α-diversity, larger β-diversity shifts, ↑ acquisition of new species, & ↓ resident taxa (partial replacement); strain engraftment confirmed.	Number of participants who will be free from tumor progression, as assessed by RECIST criteria v. 1.1	(159)
NCT04951583 (LUMINate)	Non-Small Cell Lung Cancer Metastatic, Advanced Melanoma	FMT, Healthy donors via Oral Capsules + Pembrolizumab, Ipilimumab + Nivolumab	45	R & NR: Both showed effective donor strain engraftment. Compositional shifts seen primarily in melanoma; no difference in donor-recipient similarity.	Objective response rate in the NSCLC cohort by RECIST criteria	(160)
NCT03341143	Melanoma	FMT, PD-1 responders via Colonoscopy + Pembrolizumab	20	Rapid, durable shifts; R showed stronger donor convergence, persistent engraftment, & ↑ beneficial taxa. Prevotella engraftment from rich donor linked to severe irAEs (myocarditis).	Objective Response Rate (ORR)	(106)
NCT04040712	Diarrhea, Renal Cell Cancer	FMT, Healthy donors via Colonoscopy	20	d-FMT: ↓ Bray-Curtis distance to donor, ↑ shared species/strains at weeks 1-4; robust strain engraftment up to 1 month.	rate of patients who experience resolution of diarrhea 4 weeks after the end of treatments	(161)
NCT06026371	Acute GVHD, Hematopoietic and Lymphatic System Neoplasm	FMT, Healthy donors via Oral Capsules + Allo-HCT	138	Post-allo-HCT: ↑ α-diversity; broad composition recovery toward a healthy-like donor cluster; clear donor effect on engraftment.	Grade III-IV acute graft versus host disease (GVHD)	(162)
NCT03678493	Acute Myeloid Leukemia, Allogeneic Hematopoietic Cell Transplantation	FMT via Oral Capsules + Chemotherapy/ Allo-HCT	100	Post-FMT: ↑ α-diversity, β-diversity shift toward donor microbiota clusters; ↑ obligate anaerobes; sustained donor engraftment.	Efficacy of FMT in AML Patients and Allo-HCT Recipients - Number of Infections	(163)
NCT03772899	Melanoma	FMT, Healthy donors via Oral Capsules + pembrolizumab or nivolumab	20	↑ α-diversity up to 3 months (R & NR). Only R showed stable, progressive engraftment (↑ butyrate-linked Ruminococcaceae/Blautia/Faecalibacterium, ↓ Enterocloster/Erysipelatoclostridium).	safety of combining FMT with immunotherapy in melanoma patients	(164)
NCT02928523	Leukemia, Myeloid, Acute	FMT, Individual patients/Autologous via Colonoscopy + Chemotherapy	20	Autologous FMT: Reversals of dysbiosis (↑ α-diversity & baseline similarity); restored butyrate-producing Clostridiales ecosystem; ↓ resistome expansion. Carbapenem use reduced α-diversity.	Evaluation of AFMT efficacy in dysbiosis correction by measure of microbiota diversity	(165)
NCT04163289	Renal Cell Carcinoma	FMT, Healthy donors via Capsules + ipilimumab, nivolumab	20	↑ α-diversity correlated with ↓ toxicity. R showed higher α-diversity at 10 weeks; progressive donor-like remodeling occurred mainly in R and low-toxicity patients.	Occurence of immune-related colitis associated with ipilimumab/nivolumab treatment	(166)
ChiCTR2100046768 (RENMIN-215)	Metastatic Colorectal Cancer	FMT, Healthy donors via Capsules + tislelizumab (anti−PD−1) and fruquintinib	20	↑ α−diversity and donor−like shifts with engraftment of donor taxa	Progression Free Survival (PFS); Disease Control Rate (DCR); safety	(167)
NCT05943041	Solid Tumor, Colorectal Cancer, Sigmoid Colon Cancer, Rectosigmoid Junction Cancer, Cancer, Rectal Cancer	LBPs, GB104 via oral capsules + standard treatment: curative colectomy with or without full-cycle adjuvant chemotherapy	9	Minimal changes in microbial diversity across cohorts, indicating no significant alterations of the gut microbiome.	Incidence of DLT (Dose-Limiting Toxicity) at week 4, 4 weeks	(168)
NCT05877430	NSCLC, HNSCC, Melanoma, Metastatic Cancer, Advanced Solid Tumor, Advanced Cancer	LBPs, CJRB-101 via Oral capsules + ICI with Pembrolizumab	160	↑ Abundance of supplemented therapeutic strain (successful engraftment); ↑ microbial diversity & ↑ butyrate-producing bacteria from baseline.	Tolerability and Safety; Incidence of adverse events, Assessed per CTCAE v5.0, Maximum 2 years	(169)
NCT04208958	Metastatic Cancer, Melanoma, Gastric Cancer, Gastroesophageal Junction Adenocarcinoma, Colorectal Cancer	LBPs, VE800 via Oral capsules + Immunotherapy with nivolumab	56	Robust VE800 strain colonization up to week 4; higher engraftment correlated with prolonged PFS in melanoma cohort.	Safety and Tolerability; Number of participants with adverse events from the first dose to the last dose (up to 56.7 weeks), plus 100 days of post-treatment follow-up	(170)
NCT05122546	Metastatic Renal Cell Carcinoma	LBPs, CBM588 (Bifidobacterium) via oral capsules + Immunotherapy nivolumab and cabozantinib	31	CBM588 enriched Ruminococcaceae & upregulated menaquinone biosynthesis pathways; no significant change in Bifidobacterium, α-, or β-diversity.	Change in Bifidobacterium composition of stool; Baseline to week 12 of therapy	(171)
NCT03829111	Renal cell carcinoma	Probiotics, Clostridium butyricum (CBM588) via Oral + Immunotherapy with nivolumab and ipilimumab	30	Responders: ↑ Bifidobacterium spp., ↑ SCFA pathways (propionate/butyrate), ↓ Desulfovibrio spp., ↓ E. coli, & ↓ glycolysis/pyruvate pathways. No change overall.	Determine the effect of CBM588 (in combination with nivolumab/ipilimumab) on the gut microbiome in patients with metastatic renal cell carcinoma (mRCC).	(172)
NCT03112837	Nasopharyngeal Carcinoma (NPC)	Probiotics, Lactobacillus, Bifidobacterium and Enterococcus via Oral + Radiotherapy	40	Restored Firmicutes/Bacteroidetes ratio; ↑ anti-inflammatory butyrate-producers (Lachnospiraceae, Ruminococcus), ↓ pathobionts (Bacteroides, Actinobacteria).	the incidence of Radiation Therapy Oncology Group grade 3 mucositis, confluent pseudomembranous mucosa, time frame: one year	(173)
NCT01410955/NCT02819960	Metastatic Colorectal Cancer	Probiotics, Bifidobacterium (BB-12) + L. rhamnosus (LGG) via Oral + Irinotecan	279	Mitigated overgrowth of β-glucuronidase-producing bacteria triggered by irinotecan.	Irinotecan−induced diarrhea, especially grade 3/4 (CTCAE v4.1)	(174)
ChiCTR-TRC-13003332	Colorectal Cancer Surgery	Probiotics, Three-strain medicinal capsule (Bifidobacterium, Lactobacillus, Enterococcus) via Oral Powder or gastric gavage + elective colorectal resection	60	Microbiome data not specified (primary outcomes focused on clinical improvements: improved time to flatus/defecation, ↓ postoperative diarrhea).	Bowel function and diarrheal complications post−CRC resection	(175)
NCT00936572	Colorectal Cancer Surgery	Probiotics, L. johnsonii (La1) vs B. longum (BB536) via Oral + elective colorectal resection	31	Dose-dependent colonic mucosal colonization of L. johnsonii; associated with ↓ mucosal and luminal Enterobacteriaceae.	Probiotic colonization and its effect on mucosal microflora (Enterobacteriaceae/Enterococci) and local immune cell markers (DC and T−cell subsets)	(176)
NCT06122636	Head and Neck Cancer	Probiotics, multi-strain probiotic blend via Oral + Radiotherapy	62	↓ Overall salivary bacterial burden and ↓ F. nucleatum; no broad shift in other periodontopathogens.	Changes in salivary function and oral bacterial loads after 30 days of daily probiotics versus placebo	(177)
Goto et al., 2026 (Cohort)	Non-Small Cell Lung Cancer	Probiotics, Clostridium butyricum (CBM588) via Oral + ICI	38	Microbiome composition data not specified; focused on immunomodulation (↑ frequency and activation of antitumor Vγ9Vδ2 T cells).	Activation and expansion of antitumor Vγ9Vδ2 T cells (CD69^+^ Vδ2^+^) with CBM588 + ICI	(178)
Helsinki HUH-2002 (Institutional Protocol)	Colorectal Cancer	Probiotics, Lactobacillus rhamnosus GG (LGG) via Oral + Adjuvent Chemotherapy 5-FU	150	Microbiome data not specified; focused on clinical protection (LGG reduced severe diarrhea and abdominal discomfort during 5-FU chemotherapy).	Frequency of grade 3–4 diarrhoea	(179)

5-FU, 5-Fluorouracil; Allo-HCT, allogeneic hematopoietic cell transplantation; AML, acute myeloid leukemia; Anti-PD-1, anti-programmed cell death protein 1; CRC, colorectal cancer; CTCAE, Common Terminology Criteria for Adverse Events; DC, dendritic cells; DCR, disease control rate; DLT, dose-limiting toxicity; FMT, fecal microbiota transplantation; d-FMT, donor fecal microbiota; p-FMT, placebo fecal microbiota transplantation; GVHD, graft-versus-host disease; HNSCC, head and neck squamous cell carcinoma; ICI, immune checkpoint inhibitor; irAEs, immune-related adverse events; LBP, live biotherapeutic product; mRCC, metastatic renal cell carcinoma; R, responders; NR, non-responders; NSCLC, non-small cell lung cancer; ORR, objective response rate; transplantation; PFS, progression-free survival; RECIST, Response Evaluation Criteria in Solid Tumors; SCFA, short-chain fatty acids. Symbols: ↑, increase/increased; ↓, decrease/decreased.

### Live biotherapeutic products and probiotics

LBPs are live microorganisms manufactured under Good Manufacturing Practice (GMP) standards and intended to prevent, treat or cure diseases, and are regulated like drugs, unlike supplements (183). In cancer immunotherapy, they are designed either as single strain products (e.g., *Akkermansia muciniphila*) or as multi-strain consortia enriched with taxa for improved ICI response and beneficial metabolites (184, 185). LBPs are developed as standardized, drug-grade, microbial therapies in oncology, which require IND-level dossiers, safety studies, and formal clinical trials (see **Table 1**), like other immuno-oncology agents. By contrast, most probiotics are regulated as foods or dietary supplements, not medicines. They often contain broad bacterial mixtures marketed for general gut health, with variable composition and minimal oncology-specific trial data. These off-the-shelf probiotics usually include *Lactobacillus, Lactococcus, Bifidobacterium* and *Enterococcus* (**Figure 2C**) (181, 183, 186). An observational study reported that more than 30% of melanoma patients use over−the−counter probiotics within 30 days prior to initiating immunotherapy. Although not statistically significant, probiotic users exhibited reduced progression-free survival (PFS) probabilities among patients who received ICI therapy (187). These findings prompted a cautionary note to patients and care providers regarding the potential risks associated with unsupervised use of commercial probiotics.

### Translational challenges and perspectives

The apparent discrepancies between FMT, conventional probiotics, and LBPs largely reflect how differently these microbiome-modulating strategies are designed, deployed, and measured. Across the trials summarized in **Table 1**, FMT consistently increases α-diversity, drives donor-like compositional shifts, and achieves strain-level engraftment in diverse contexts including advanced melanoma, RCC, NSCLC, allo-HCT, ICI-induced colitis, and chemotherapy-associated dysbiosis. However, the associated clinical benefits are heterogeneous: some early melanoma and RCC studies suggest meaningful gains in ICI responsiveness or toxicity control, whereas infection prevention, aGVHD mitigation, and diarrhea resolution show more modest or context-dependent effects in small, often non-randomized or early-phase trials. These data support a view of FMT as a potent, but indication- and design-dependent adjuvant, rather than a universally effective immuno-oncology tool.

A similar picture emerges for probiotics and LBPs. Conventional probiotics in oncology typically involve empiric, multi-strain commercial mixtures administered to broad, often unselected patient populations, with endpoints focused on gastrointestinal toxicity or perioperative recovery. Such products frequently modulate gut or oral communities, normalizing dysbiotic phylum-level patterns, enriching butyrate-producing *Lachnospiraceae/Ruminococcaceae* or *Bifidobacterium*, and reducing pathobionts such as *Enterobacteriaceae*, β-glucuronidase-producers and *F. nucleatum*. Yet their impact on hard oncologic outcomes (for example, progression-free or overall survival) is modest, inconsistent, or not assessed, and several trials with clear clinical benefit did not include microbiome profiling. By contrast, LBPs such as CBM588, VE800, CJRB-101 and GB104 are strain-defined consortia manufactured under GMP conditions and purpose-built for specific clinical settings (for example, ICI-treated mRCC or NSCLC). These products reproducibly engraft and induce targeted functional shifts (for instance, in SCFA or menaquinone biosynthesis), and some early-phase studies report encouraging improvements in ICI response and progression-free survival in carefully selected cohorts, even as other formulations show predominantly microbiological, rather than clearly robust, clinical effects.

Taken together, the FMT, probiotic and LBP trials (**Table 1**) argue that microbiome-based interventions are highly context-dependent rather than intrinsically inconsistent. Outcomes vary with tumor type and treatment backbone (chemotherapy, radiotherapy, allo-HCT, ICI), with intervention design (whole-stool FMT versus empiric probiotics versus rationally engineered consortia), and with trial features such as donor/source, antibiotic preconditioning, dosing schedules, and sample size. In parallel, proposed microbiome biomarkers of treatment response remain unstable across cohorts: the specific “favorable” taxa and pathways differ between melanoma, RCC, NSCLC and perioperative settings, and reproducibility is further undermined by heterogeneity in sampling and sequencing methods and by host factors such as diet and antibiotic exposure. These observations argue that FMT, probiotics and LBPs should all be evaluated within indication-specific, mechanistically informed frameworks, with harmonized microbiome methodology and prospective biomarker development. Within this framework, FMT and LBPs can be viewed as complementary steps toward microbiome-driven precision medicine, where defined or engineered consortia, and selected donor communities, are co-developed with ICIs and other immunotherapies.

## Challenges and future perspectives

### Challenges

Challenges in diagnostics and personalized therapy for cancer treatment persist despite advances in microbiome profiling and AI-driven patient stratification. Challenges evolve primarily around standardization, reproducibility, and clinical translation. In addition, inter- and intra-tumor heterogeneity complicates biomarker validation, as microbiome signatures can vary substantially depending on geography, diet, and analytical approaches (16S rRNA gene sequencing versus metagenomics). This variability leads to inconsistent sensitivity and specificity across patient cohorts. Moreover, longitudinal monitoring of dysbiosis remains challenging due to differences in sampling strategies and depth (repeated stool versus biopsy collections) as well as the limited availability of reference databases for real-time identification of pathogens that negatively impact therapy outcomes (188).

Personalized microbiome modulations, including FMT and probiotics, face substantial translational barriers. These include scalability constraints, regulatory challenges surrounding the commercialization and use of LBPs, and unpredictable microbial engraftment, with evidence showing that only a minority of immunotherapy non-responders derive benefit from these interventions. In parallel, drug-microbiome interactions may introduce unintended effects, such as depletion of beneficial taxa during combination therapies or selection for antimicrobial-resistant organisms. More broadly, efforts to translate microbiome- and diet-based strategies into clinical practice are hindered by significant methodological and analytical limitations. Experimental studies in animal models often rely on non-standardized or nutritionally unbalanced diets that inadequately reflect human consumption patterns, while human trials are limited by small cohorts, short durations, and poor dietary control. Additional challenges include patient compliance, recall bias in dietary assessments and technical variability in sampling and multi-omics profiling. Although emerging omics approaches, including integrated microbiome, genomic and dietary exposome analyses, offer more objective and comprehensive insights into host-microbe interactions, their implementation in routine care remains constrained by high costs, computational complexity, and the need for prospective validation (189). Addressing these challenges requires standardized, scalable multi-omics workflows, improved reference frameworks, and harmonized regulatory pathways to enable the clinical translation of microbiome-informed and nutrition-based precision strategies (188).

### Future perspectives

In the years ahead, translating microbiome discoveries into robust cancer diagnostics and personalized therapy will depend on overcoming current challenges in standardization, reproducibility, and clinical implementation. To accomplish this, the field is moving toward harmonized multi−omics frameworks that integrate high−resolution microbiome profiling (e.g., shotgun metagenomics) with host genomics, transcriptomics, metabolomics, and immune−phenotyping into unified, AI−driven models for early detection and prediction of therapy response (190, 191). Machine learning approaches such as random forests and XGBoost enable multi-modal data fusion and identification of composite predictive signatures.

Fecal metagenomics has identified enrichment of taxa such as *Bifidobacterium* and *Faecalibacterium*, while metabolomics highlights short-chain fatty acids and bile acid derivatives, and transcriptomic profiling captures interferon-γ and PD-L1–associated immune activation (192, 193). Late-fusion models integrating these features have shown moderate-to-high predictive performance across melanoma, colorectal cancer, and non-small cell lung cancer cohorts, although generalizability remains limited by cohort heterogeneity and small sample sizes (194).

Key challenges include the “small N, large P” problem, batch effects across sequencing platforms, and lack of standardized multi-omics pipelines. These issues limit reproducibility and clinical translation. From a computational perspective, explainable AI approaches such as SHapley Additive exPlanations (SHAP) improve interpretability by identifying biologically relevant predictors, while federated learning enables multi-institutional model training without centralized data sharing, preserving privacy and reducing inter-cohort variability (195). Together with standardized workflows (e.g., QIIME2), these advances are essential for developing robust and clinically deployable microbiome-informed predictive models (196, 197).

Longitudinal monitoring of microbiome dynamics during treatment represents another critical step. Serial sampling approaches (e.g., stool or minimally invasive biopsies) coupled with real−time bioinformatic pipelines, could enable tracking of dysbiosis, drug−induced perturbations, and microbiome−drug interactions, ultimately supporting adaptive interventions such as dietary modulation or targeted microbial therapies. At the therapeutic level, the field is expected to move beyond conventional probiotics and unrefined FMT toward rationally designed microbial consortia and engineered “smart” microbes. These next-generation approaches aim to deliver immunomodulatory or chemoprotective functions in a tumor− or patient−specific manner, while minimizing off−target effects and engraftment consistency (198, 199).

Finally, successful integration into routine oncology will require not only standardized assays and analytical pipelines, but also clear regulatory frameworks for microbiome−based tests and live biotherapeutics. Prospective randomized trials testing predefined microbiome−guided strategies (e.g., microbiome−stratified immunotherapy or microbiome−preserving antibiotic regimens) will be needed to demonstrate clinical benefit over current standards of care. With coordinated efforts across microbiome science, oncology, bioinformatics, and regulatory science, microbiome−informed precision oncology has the potential to evolve from a research concept into a clinically actionable component of cancer care.

## References

[1] Gill SR Pop M Deboy RT Eckburg PB Turnbaugh PJ Samuel BS . Metagenomic analysis of the human distal gut microbiome. Science. (2006) 312:1355–9. doi: 10.1126/science.1124234 16741115 PMC3027896

[2] den Besten G van Eunen K Groen AK Venema K Reijngoud DJ Bakker BM . The role of short-chain fatty acids in the interplay between diet, gut microbiota, and host energy metabolism. J Lipid Res. (2013) 54:2325–40. doi: 10.1194/jlr.R036012 23821742 PMC3735932

[3] Gensollen T Iyer SS Kasper DL Blumberg RS . How colonization by microbiota in early life shapes the immune system. Science. (2016) 352:539–44. doi: 10.1126/science.aad9378 27126036 PMC5050524

[4] Bäumler AJ Sperandio V . Interactions between the microbiota and pathogenic bacteria in the gut. Nature. (2016) 535:85–93. doi: 10.1038/nature18849 27383983 PMC5114849

[5] DeGruttola AK Low D Mizoguchi A Mizoguchi E . Current understanding of dysbiosis in disease in human and animal models. Inflammation Bowel Dis. (2016) 22:1137–50. doi: 10.1097/MIB.0000000000000750 27070911 PMC4838534

[6] Leone V Chang EB Devkota S . Diet, microbes, and host genetics: the perfect storm in inflammatory bowel diseases. J Gastroenterol. (2013) 48:315–21. doi: 10.1007/s00535-013-0777-2 23475322 PMC3698420

[7] Gopalakrishnan V Spencer CN Nezi L Reuben A Andrews MC Karpinets TV . Gut microbiome modulates response to anti-PD-1 immunotherapy in melanoma patients. Science. (2018) 359:97–103. doi: 10.1126/science.aan4236 29097493 PMC5827966

[8] Cuevas-Ramos G Petit CR Marcq I Boury M Oswald E Nougayrède JP . Escherichia coli induces DNA damage *in vivo* and triggers genomic instability in mammalian cells. Proc Natl Acad Sci USA. (2010) 107:11537–42. doi: 10.1073/pnas.1001261107 20534522 PMC2895108

[9] Tjalsma H Boleij A Marchesi JR Dutilh BE . A bacterial driver-passenger model for colorectal cancer: beyond the usual suspects. Nat Rev Microbiol. (2012) 10:575–82. doi: 10.1038/nrmicro2819 22728587

[10] Jones J Shi Q Nath RR Brito IL . Keystone pathobionts associated with colorectal cancer promote oncogenic reprograming. bioRxiv. (2023) 19(2):e0297897. doi: 10.1101/2023.04.03.535410. Preprint. 38363784 PMC10871517

[11] Skinner GR . Transformation of primary hamster embryo fibroblasts by type 2 simplex virus: evidence for a "hit and run" mechanism. Br J Exp Pathol. (1976) 57:361–76. PMC2041152 183803

[12] Qin J Li R Raes J Arumugam M Burgdorf KS Manichanh C . A human gut microbial gene catalogue established by metagenomic sequencing. Nature. (2010) 464:59–65. doi: 10.1038/nature08821 20203603 PMC3779803

[13] Desai MS Seekatz AM Koropatkin NM Kamada N Hickey CA Wolter M . A dietary fiber-deprived gut microbiota degrades the colonic mucus barrier and enhances pathogen susceptibility. Cell. (2016) 167:1339–1353.e21. doi: 10.1016/j.cell.2016.10.043 27863247 PMC5131798

[14] Carmody RN Gerber GK Luevano JM Gatti DM Somes L Svenson KL . Diet dominates host genotype in shaping the murine gut microbiota. Cell Host Microbe. (2015) 17:72–84. doi: 10.1016/j.chom.2014.11.010 25532804 PMC4297240

[15] Yang M Zheng X Fan J Cheng W Yan TM Lai Y . Antibiotic-induced gut microbiota dysbiosis modulates host transcriptome and m6A epitranscriptome via bile acid metabolism. Adv Sci (Weinh). (2024) 11:e2307981. doi: 10.1002/advs.202307981 38713722 PMC11267274

[16] Hrncir T . Gut microbiota dysbiosis: triggers, consequences, diagnostic and therapeutic options. Microorganisms. (2022) 10:578. doi: 10.3390/microorganisms10030578 35336153 PMC8954387

[17] Park SH Choi U Ryu SH Lee HB Lee JW Lee CR . Divergent effects of peptidoglycan carboxypeptidase DacA on intrinsic β-lactam and vancomycin resistance. Microbiol Spectr. (2022) 10:e0173422. doi: 10.1128/spectrum.01734-22 35758683 PMC9430164

[18] Ubeda C Taur Y Jenq RR Equinda MJ Son T Samstein M . Vancomycin-resistant Enterococcus domination of intestinal microbiota is enabled by antibiotic treatment in mice and precedes bloodstream invasion in humans. J Clin Invest. (2010) 120:4332–41. doi: 10.1172/JCI43918 21099116 PMC2993598

[19] Dethlefsen L Huse S Sogin ML Relman DA . The pervasive effects of an antibiotic on the human gut microbiota, as revealed by deep 16S rRNA sequencing. PloS Biol. (2008) 6:e280. doi: 10.1371/journal.pbio.0060280 19018661 PMC2586385

[20] Rea MC Dobson A O'Sullivan O Crispie F Fouhy F Cotter PD . Effect of broad- and narrow-spectrum antimicrobials on Clostridium difficile and microbial diversity in a model of the distal colon. Proc Natl Acad Sci USA. (2011) 108 Suppl 1:4639–44. doi: 10.1073/pnas.1001224107 20616009 PMC3063588

[21] Dörner PJ Anandakumar H Röwekamp I Fiocca Vernengo F Millet Pascual-Leone B Krzanowski M . Clinically used broad-spectrum antibiotics compromise inflammatory monocyte-dependent antibacterial defense in the lung. Nat Commun. (2024) 15:2788. doi: 10.1038/s41467-024-47149-z 38555356 PMC10981692

[22] Francino MP . Antibiotics and the human gut microbiome: dysbioses and accumulation of resistances. Front Microbiol. (2016) 6:1543. doi: 10.3389/fmicb.2015.01543 26793178 PMC4709861

[23] Patangia DV Anthony Ryan C Dempsey E Paul Ross R Stanton C . Impact of antibiotics on the human microbiome and consequences for host health. Microbiol Open. (2022) 11:e1260. doi: 10.1002/mbo3.1260 35212478 PMC8756738

[24] Ramirez J Guarner F Bustos Fernandez L Maruy A Sdepanian VL Cohen H . Antibiotics as major disruptors of gut microbiota. Front Cell Infect Microbiol. (2020) 10:572912. doi: 10.3389/fcimb.2020.572912 33330122 PMC7732679

[25] Frank DN St Amand AL Feldman RA Boedeker EC Harpaz N Pace NR . Molecular-phylogenetic characterization of microbial community imbalances in human inflammatory bowel diseases. Proc Natl Acad Sci USA. (2007) 104:13780–5. doi: 10.1073/pnas.0706625104 17699621 PMC1959459

[26] Karlsson FH Tremaroli V Nookaew I Bergström G Behre CJ Fagerberg B . Gut metagenome in European women with normal, impaired and diabetic glucose control. Nature. (2013) 498:99–103. doi: 10.1038/nature12198 23719380

[27] Abrahamsson TR Jakobsson HE Andersson AF Björkstén B Engstrand L Jenmalm MC . Low gut microbiota diversity in early infancy precedes asthma at school age. Clin Exp Allergy. (2014) 44:842–50. doi: 10.1111/cea.12253 24330256

[28] Petersen C Round JL . Defining dysbiosis and its influence on host immunity and disease. Cell Microbiol. (2014) 16:1024–33. doi: 10.1111/cmi.12308 24798552 PMC4143175

[29] Sun M Wu W Chen L Yang W Huang X Ma C . Microbiota-derived short-chain fatty acids promote Th1 cell IL-10 production to maintain intestinal homeostasis. Nat Commun. (2018) 9:3555. doi: 10.1038/s41467-018-05901-2 30177845 PMC6120873

[30] Greco G Zeppa SD Agostini D Attisani G Stefanelli C Ferrini F . The anti- and pro-tumorigenic role of microbiota and its role in anticancer therapeutic strategies. Cancers (Basel). (2022) 15:190. doi: 10.3390/cancers15010190 36612186 PMC9818275

[31] Takeuchi O Akira S . Pattern recognition receptors and inflammation. Cell. (2010) 140:805–20. doi: 10.1016/j.cell.2010.01.022 20303872

[32] Sun X Liu L Wang J Luo X Wang M Wang C . Targeting STING in dendritic cells alleviates psoriatic inflammation by suppressing IL-17A production. Cell Mol Immunol. (2024) 21:738–51. doi: 10.1038/s41423-024-01160-y 38806624 PMC11214627

[33] Ashkenazi-Preiser H Reuven O Uzan-Yulzari A Komisarov S Cirkin R Turjeman S . The cross-talk between intestinal microbiota and MDSCs fuels colitis-associated cancer development. Cancer Res Commun. (2024) 4:1063–81. doi: 10.1158/2767-9764.CRC-23-0421 38506672 PMC11017962

[34] Gur C Maalouf N Shhadeh A Berhani O Singer BB Bachrach G . Fusobacterium nucleatum supresses anti-tumor immunity by activating CEACAM1. Oncoimmunology. (2019) 8:e1581531. doi: 10.1080/2162402X.2019.1581531 31069151 PMC6492956

[35] Guo P Tian Z Kong X Yang L Shan X Dong B . FadA promotes DNA damage and progression of Fusobacterium nucleatum-induced colorectal cancer through up-regulation of chk2. J Exp Clin Cancer Res. (2020) 39:202. doi: 10.1186/s13046-020-01677-w 32993749 PMC7523382

[36] Le DT Uram JN Wang H Bartlett BR Kemberling H Eyring AD . PD-1 blockade in tumors with mismatch-repair deficiency. N Engl J Med. (2015) 372:2509–20. doi: 10.1056/NEJMoa1500596 26028255 PMC4481136

[37] Llosa NJ Cruise M Tam A Wicks EC Hechenbleikner EM Taube JM . The vigorous immune microenvironment of microsatellite instable colon cancer is balanced by multiple counter-inhibitory checkpoints. Cancer Discov. (2015) 5:43–51. doi: 10.1158/2159-8290.CD-14-0863 25358689 PMC4293246

[38] Mima K Cao Y Chan AT Qian ZR Nowak JA Masugi Y . Fusobacterium nucleatum in colorectal carcinoma tissue according to tumor location. Clin Transl Gastroenterol. (2016) 7:e200. doi: 10.1038/ctg.2016.53 27811909 PMC5543402

[39] Yu T Guo F Yu Y Sun T Ma D Han J . Fusobacterium nucleatum promotes chemoresistance to colorectal cancer by modulating autophagy. Cell. (2017) 170:548–563.e16. doi: 10.1016/j.cell.2017.07.008 28753429 PMC5767127

[40] Overman MJ McDermott R Leach JL Lonardi S Lenz HJ Morse MA . Nivolumab in patients with metastatic DNA mismatch repair-deficient or microsatellite instability-high colorectal cancer (CheckMate 142): an open-label, multicentre, phase 2 study. Lancet Oncol. (2017) 18:1182–91. doi: 10.1016/S1470-2045(17)30422-9 28734759 PMC6207072

[41] Chen T Li Q Wu J Wu Y Peng W Li H . Fusobacterium nucleatum promotes M2 polarization of macrophages in the microenvironment of colorectal tumours via a TLR4-dependent mechanism. Cancer Immunol Immunother. (2018) 67:1635–46. doi: 10.1007/s00262-018-2233-x 30121899 PMC11028377

[42] Hu L Liu Y Kong X Wu R Peng Q Zhang Y . Fusobacterium nucleatum facilitates M2 macrophage polarization and colorectal carcinoma progression by activating TLR4/NF-κB/S100A9 cascade. Front Immunol. (2021) 12:658681. doi: 10.3389/fimmu.2021.658681 34093546 PMC8176789

[43] Gao Y Bi D Xie R Li M Guo J Liu H . Fusobacterium nucleatum enhances the efficacy of PD-L1 blockade in colorectal cancer. Signal Transduct Target Ther. (2021) 6:398. doi: 10.1038/s41392-021-00795-x 34795206 PMC8602417

[44] Kim HS Kim CG Kim WK Kim KA Yoo J Min BS . Fusobacterium nucleatum induces a tumor microenvironment with diminished adaptive immunity against colorectal cancers. Front Cell Infect Microbiol. (2023) 13:1101291. doi: 10.3389/fcimb.2023.1101291 36960042 PMC10028079

[45] Zhang T Li Y Zhai E Zhao R Qian Y Huang Z . Intratumoral Fusobacterium nucleatum recruits tumor-associated neutrophils to promote gastric cancer progression and immune evasion. Cancer Res. (2025) 85:1819–41. doi: 10.1158/0008-5472.CAN-24-2580 39992708 PMC12079103

[46] Ye C Liu X Liu Z Pan C Zhang X Zhao Z . Fusobacterium nucleatum in tumors: from tumorigenesis to tumor metastasis and tumor resistance. Cancer Biol Ther. (2024) 25:2306676. doi: 10.1080/15384047.2024.2306676 38289287 PMC10829845

[47] Parhi L Alon-Maimon T Sol A Nejman D Shhadeh A Fainsod-Levi T . Breast cancer colonization by Fusobacterium nucleatum accelerates tumor growth and metastatic progression. Nat Commun. (2020) 11:3259. doi: 10.1038/s41467-020-16967-2 32591509 PMC7320135

[48] Pelaseyed T Bergström JH Gustafsson JK Ermund A Birchenough GM Schütte A . The mucus and mucins of the goblet cells and enterocytes provide the first defense line of the gastrointestinal tract and interact with the immune system. Immunol Rev. (2014) 260:8–20. doi: 10.1111/imr.12182 24942678 PMC4281373

[49] Laukoetter MG Nava P Lee WY Severson EA Capaldo CT Babbin BA . JAM-A regulates permeability and inflammation in the intestine *in vivo*. J Exp Med. (2007) 204:3067–76. doi: 10.1084/jem.20071416 18039951 PMC2150975

[50] Ouellette AJ Bevins CL . Paneth cell defensins and innate immunity of the small bowel. Inflammation Bowel Dis. (2001) 7:43–50. doi: 10.1097/00054725-200102000-00007 11233660

[51] Wehkamp J Fellermann K Herrlinger KR Bevins CL Stange EF . Mechanisms of disease: defensins in gastrointestinal diseases. Nat Clin Pract Gastroenterol Hepatol. (2005) 2:406–15. doi: 10.1038/ncpgasthep0265 16265431

[52] Wehkamp J Salzman NH Porter E Nuding S Weichenthal M Petras RE . Reduced Paneth cell alpha-defensins in ileal Crohn's disease. Proc Natl Acad Sci USA. (2005) 102:18129–34. doi: 10.1073/pnas.0505256102 16330776 PMC1306791

[53] Pagel R Bär F Schröder T Sünderhauf A Künstner A Ibrahim SM . Circadian rhythm disruption impairs tissue homeostasis and exacerbates chronic inflammation in the intestine. FASEB J. (2017) 31:4707–19. doi: 10.1096/fj.201700141RR 28710114 PMC6159707

[54] Wu S Lim KC Huang J Saidi RF Sears CL . Bacteroides fragilis enterotoxin cleaves the zonula adherens protein, E-cadherin. Proc Natl Acad Sci USA. (1998) 95:14979–84. doi: 10.1073/pnas.95.25.14979 9844001 PMC24561

[55] Yang J Wang X Hu T Huang H Chen G Jin B . Entero-toxigenic Bacteroides fragilis contributes to intestinal barrier injury and colorectal cancer progression by mediating the BFT/STAT3/ZEB2 pathway. Cell Cycle. (2024) 23:70–82. doi: 10.1080/15384101.2024.2309005 38273425 PMC11005799

[56] Steck N Hoffmann M Sava IG Kim SC Hahne H Tonkonogy SL . Enterococcus faecalis metalloprotease compromises epithelial barrier and contributes to intestinal inflammation. Gastroenterology. (2011) 141:959–71. doi: 10.1053/j.gastro.2011.05.035 21699778

[57] Belogortseva N Krezalek M Guyton K Labno C Poroyko V Zaborina O . Media from macrophages co-incubated with Enterococcus faecalis induces epithelial cell monolayer reassembly and altered cell morphology. PloS One. (2017) 12:e0182825. doi: 10.1371/journal.pone.0182825 28793333 PMC5549984

[58] Vizcaino MI Crawford JM . The colibactin warhead crosslinks DNA. Nat Chem. (2015) 7:411–7. doi: 10.1038/nchem.2221 25901819 PMC4499846

[59] Chaturvedi R Asim M Piazuelo MB Yan F Barry DP Sierra JC . Activation of EGFR and ERBB2 by Helicobacter pylori results in survival of gastric epithelial cells with DNA damage. Gastroenterology. (2014) 146:1739–53. doi: 10.1053/j.gastro.2014.02.005 24530706 PMC4035375

[60] Auvray F Perrat A Arimizu Y Chagneau CV Bossuet-Greif N Massip C . Insights into the acquisition of the pks island and production of colibactin in the Escherichia coli population. Microb Genom. (2021) 7:579. doi: 10.1099/mgen.0.000579 33961542 PMC8209727

[61] Pleguezuelos-Manzano C Puschhof J Rosendahl Huber A van Hoeck A Wood HM Nomburg J . Mutational signature in colorectal cancer caused by genotoxic pks+ E. coli. Nature. (2020) 580:269–73. doi: 10.1038/s41586-020-2080-8 32106218 PMC8142898

[62] Volpe MR Velilla JA Daniel-Ivad M Yao JJ Stornetta A Villalta PW . A small molecule inhibitor prevents gut bacterial genotoxin production. Nat Chem Biol. (2023) 19:159–67. doi: 10.1038/s41589-022-01147-8 36253549 PMC9889270

[63] Han T Jing X Bao J Zhao L Zhang A Miao R . H. pylori infection alters repair of DNA double-strand breaks via SNHG17. J Clin Invest. (2020) 130:3901–18. doi: 10.1172/JCI125581 32538894 PMC7324211

[64] Chu H Williams B Schnabl B . Gut microbiota, fatty liver disease, and hepatocellular carcinoma. Liver Res. (2018) 2:43–51. doi: 10.1016/j.livres.2017.11.005 30416839 PMC6223644

[65] Mahboob A Shin C Almughanni S Hornakova L Kubatka P Büsselberg D . The gut nexus: unraveling microbiota-mediated links between type 2 diabetes and colorectal cancer. Nutrients. (2025) 17:3803. doi: 10.3390/nu17233803 41374093 PMC12694093

[66] Said I Ahad H Said A . Gut microbiome in non-alcoholic fatty liver disease associated hepatocellular carcinoma: Current knowledge and potential for therapeutics. World J Gastrointest Oncol. (2022) 14:947–58. doi: 10.4251/wjgo.v14.i5.947 35646285 PMC9124992

[67] Kant P Hull MA . Excess body weight and obesity--the link with gastrointestinal and hepatobiliary cancer. Nat Rev Gastroenterol Hepatol. (2011) 8:224–38. doi: 10.1038/nrgastro.2011.23 21386810

[68] Afshin A Forouzanfar MH Reitsma MB Sur P Estep K Lee A . Health effects of overweight and obesity in 195 countries over 25 years. N Engl J Med. (2017) 377:13–27. doi: 10.1056/NEJMoa1614362 28604169 PMC5477817

[69] Must A Spadano J Coakley EH Field AE Colditz G Dietz WH . The disease burden associated with overweight and obesity. JAMA. (1999) 282:1523–9. doi: 10.1001/jama.282.16.1523 10546691

[70] Zhang X Coker OO Chu ES Fu K Lau HCH Wang YX . Dietary cholesterol drives fatty liver-associated liver cancer by modulating gut microbiota and metabolites. Gut. (2021) 70:761–74. doi: 10.1136/gutjnl-2019-319664 32694178 PMC7948195

[71] Nunez N Derré-Bobillot A Trainel N Lakisic G Lecomte A Mercier-Nomé F . The unforeseen intracellular lifestyle of Enterococcus faecalis in hepatocytes. Gut Microbes. (2022) 14:2058851. doi: 10.1080/19490976.2022.2058851 35373699 PMC8986240

[72] Iida N Mizukoshi E Yamashita T Yutani M Seishima J Wang Z . Chronic liver disease enables gut Enterococcus faecalis colonization to promote liver carcinogenesis. Nat Cancer. (2021) 2:1039–54. doi: 10.1038/s43018-021-00251-3 35121877

[73] Maharshak N Huh EY Paiboonrungruang C Shanahan M Thurlow L Herzog J . Enterococcus faecalis Gelatinase Mediates Intestinal Permeability via Protease-Activated Receptor 2. Infect Immun. (2015) 83:2762–70. doi: 10.1128/IAI.00425-15 25916983 PMC4468563

[74] Lang S Demir M Duan Y Martin A Schnabl B . Cytolysin-positive Enterococcus faecalis is not increased in patients with non-alcoholic steatohepatitis. Liver Int. (2020) 40:860–5. doi: 10.1111/liv.14377 31943701 PMC8026328

[75] Wang L Fouts DE Stärkel P Hartmann P Chen P Llorente C . Intestinal REG3 lectins protect against alcoholic steatohepatitis by reducing mucosa-associated microbiota and preventing bacterial translocation. Cell Host Microbe. (2016) 19:227–39. doi: 10.1016/j.chom.2016.01.003 26867181 PMC4786170

[76] Duan Y Llorente C Lang S Brandl K Chu H Jiang L . Bacteriophage targeting of gut bacterium attenuates alcoholic liver disease. Nature. (2019) 575:505–11. doi: 10.1038/s41586-019-1742-x 31723265 PMC6872939

[77] Van Tyne D Martin MJ Gilmore MS . Structure, function, and biology of the Enterococcus faecalis cytolysin. Toxins (Basel). (2013) 5:895–911. doi: 10.3390/toxins5050895 23628786 PMC3709268

[78] Yang Y Hartmann P Schnabl B . Fecal gelatinase does not predict mortality in patients with alcohol-associated hepatitis. Microb Cell. (2024) 11:328–38. doi: 10.15698/mic2024.08.836 39206205 PMC11350238

[79] Ma N Xie X Wang J Zheng Z Jin H Chen X . Enterococcus faecalis delivers Obg GTPase via extracellular vesicles to instigate mTOR activity and promote HCC tumorigenesis. In: Biorxiv (2025). Cold Spring Harbor, NY, USA: BioRxi p. 2025.09.30.653999. doi: 10.1101/2025.09.30.653999

[80] Wong SH Yu J . Gut microbiota in colorectal cancer: mechanisms of action and clinical applications. Nat Rev Gastroenterol Hepatol. (2019) 16:690–704. doi: 10.1038/s41575-019-0209-8 31554963

[81] Mei S Deng Z Chen Y Ning D Guo Y Fan X . Dysbiosis: The first hit for digestive system cancer. Front Physiol. (2022) 13:1040991. doi: 10.3389/fphys.2022.1040991 36483296 PMC9723259

[82] Irrazabal T Thakur BK Kang M Malaise Y Streutker C Wong EOY . Limiting oxidative DNA damage reduces microbe-induced colitis-associated colorectal cancer. Nat Commun. (2020) 11:1802. doi: 10.1038/s41467-020-15549-6 32286276 PMC7156452

[83] Pushalkar S Hundeyin M Daley D Zambirinis CP Kurz E Mishra A . The pancreatic cancer microbiome promotes oncogenesis by induction of innate and adaptive immune suppression. Cancer Discov. (2018) 8:403–16. doi: 10.1158/2159-8290.CD-17-1134 29567829 PMC6225783

[84] McAllister F Khan MAW Helmink B Wargo JA . The tumor microbiome in pancreatic cancer: bacteria and beyond. Cancer Cell. (2019) 36:577–9. doi: 10.1016/j.ccell.2019.11.004 31951558

[85] Isherwood J Arshad A Chung WY Runau F Cooke J Pollard C . Myeloid derived suppressor cells are reduced and T regulatory cells stabilised in patients with advanced pancreatic cancer treated with gemcitabine and intravenous omega 3. Ann Transl Med. (2020) 8:172. doi: 10.21037/atm.2020.02.02 32309319 PMC7154395

[86] Clark CE Hingorani SR Mick R Combs C Tuveson DA Vonderheide RH . Dynamics of the immune reaction to pancreatic cancer from inception to invasion. Cancer Res. (2007) 67:9518–27. doi: 10.1158/0008-5472.CAN-07-0175 17909062

[87] Marstrand-Daucé L Lorenzo D Chassac A Nicole P Couvelard A Haumaitre C . Acinar-to-ductal metaplasia (ADM): on the road to pancreatic intraepithelial neoplasia (PanIN) and pancreatic cancer. Int J Mol Sci. (2023) 24:9946. doi: 10.3390/ijms24129946 37373094 PMC10298625

[88] Liu L Wang K Zhu ZM Shao JH . Associations between P53 Arg72Pro and development of digestive tract cancers: a meta-analysis. Arch Med Res. (2011) 42:60–9. doi: 10.1016/j.arcmed.2011.01.008 21376265

[89] Thomas RM Gharaibeh RZ Gauthier J Beveridge M Pope JL Guijarro MV . Intestinal microbiota enhances pancreatic carcinogenesis in preclinical models. Carcinogenesis. (2018) 39:1068–78. doi: 10.1093/carcin/bgy073 29846515 PMC6067127

[90] Sanapareddy N Legge RM Jovov B McCoy A Burcal L Araujo-Perez F . Increased rectal microbial richness is associated with the presence of colorectal adenomas in humans. ISME J. (2012) 6:1858–68. doi: 10.1038/ismej.2012.43 22622349 PMC3446812

[91] Lee CG Hwang S Gwon SY Park C Jo M Hong JE . Bacteroides fragilis toxin induces intestinal epithelial cell secretion of interleukin-8 by the E-cadherin/β-catenin/NF-κB dependent pathway. Biomedicines. (2022) 10:827. doi: 10.3390/biomedicines10040827 35453577 PMC9032310

[92] Shiryaev SA Remacle AG Chernov AV Golubkov VS Motamedchaboki K Muranaka N . Substrate cleavage profiling suggests a distinct function of Bacteroides fragilis metalloproteinases (fragilysin and metalloproteinase II) at the microbiome-inflammation-cancer interface. J Biol Chem. (2013) 288:34956–67. doi: 10.1074/jbc.M113.516153 24145028 PMC3843106

[93] Boleij A Hechenbleikner EM Goodwin AC Badani R Stein EM Lazarev MG . The Bacteroides fragilis toxin gene is prevalent in the colon mucosa of colorectal cancer patients. Clin Infect Dis. (2015) 60:208–15. doi: 10.1093/cid/ciu787 25305284 PMC4351371

[94] Wang T Cai G Qiu Y Fei N Zhang M Pang X . Structural segregation of gut microbiota between colorectal cancer patients and healthy volunteers. ISME J. (2012) 6:320–9. doi: 10.1038/ismej.2011.109 21850056 PMC3260502

[95] Belcheva A Irrazabal T Robertson SJ Streutker C Maughan H Rubino S . Gut microbial metabolism drives transformation of MSH2-deficient colon epithelial cells. Cell. (2014) 158:288–99. doi: 10.1016/j.cell.2014.04.051 25036629

[96] Cao Y Oh J Xue M Huh WJ Wang J Gonzalez-Hernandez JA . Commensal microbiota from patients with inflammatory bowel disease produce genotoxic metabolites. Science. (2022) 378:eabm3233. doi: 10.1126/science.abm3233 36302024 PMC9993714

[97] Pierrard J Seront E . Impact of the gut microbiome on immune checkpoint inhibitor efficacy-a systematic review. Curr Oncol. (2019) 26:395–403. doi: 10.3747/co.26.5177 31896938 PMC6927774

[98] Tanoue T Morita S Plichta DR Skelly AN Suda W Sugiura Y . A defined commensal consortium elicits CD8 T cells and anti-cancer immunity. Nature. (2019) 565:600–5. doi: 10.1038/s41586-019-0878-z 30675064

[99] Lu Y Yuan H Liang S Li D Jiang P Wang X . Microbial metabolite-driven immune reprogramming in tumor immunotherapy: mechanisms and therapeutic perspectives. Front Immunol. (2025) 16:1603658. doi: 10.3389/fimmu.2025.1603658 41181090 PMC12575249

[100] Yang Y Ye M Song Y Xing W Zhao X Li Y . Gut microbiota and SCFAs improve the treatment efficacy of chemotherapy and immunotherapy in NSCLC. NPJ Biofilms Microbiomes. (2025) 11:146. doi: 10.1038/s41522-025-00785-9 40721426 PMC12304354

[101] Luu M Riester Z Baldrich A Reichardt N Yuille S Busetti A . Microbial short-chain fatty acids modulate CD8+ T cell responses and improve adoptive immunotherapy for cancer. Nat Commun. (2021) 12:4077. doi: 10.1038/s41467-021-24331-1 34210970 PMC8249424

[102] Crespin A Le Bescop C de Gunzburg J Vitry F Zalcman G Cervesi J . A systematic review and meta-analysis evaluating the impact of antibiotic use on the clinical outcomes of cancer patients treated with immune checkpoint inhibitors. Front Oncol. (2023) 13:1075593. doi: 10.3389/fonc.2023.1075593 36937417 PMC10019357

[103] Zhao Q Chen Y Huang W Zhou H Zhang W . Drug-microbiota interactions: an emerging priority for precision medicine. Signal Transduct Target Ther. (2023) 8:386. doi: 10.1038/s41392-023-01619-w 37806986 PMC10560686

[104] Stein-Thoeringer CK Saini NY Zamir E Blumenberg V Schubert ML Mor U . A non-antibiotic-disrupted gut microbiome is associated with clinical responses to CD19-CAR-T cell cancer immunotherapy. Nat Med. (2023) 29:906–16. doi: 10.1038/s41591-023-02234-6 36914893 PMC10121864

[105] Tao M Xue M Zhou D Zhang L Hou X Zhu X . Lipopolysaccharide induces resistance to CAR-T cell therapy of colorectal cancer cells through TGF-β-mediated stemness enhancement. Mol Pharm. (2025) 22:1790–803. doi: 10.1021/acs.molpharmaceut.4c00264 40116228

[106] Davar D Dzutsev AK McCulloch JA Rodrigues RR Chauvin JM Morrison RM . Fecal microbiota transplant overcomes resistance to anti-PD-1 therapy in melanoma patients. Science. (2021) 371:595–602. doi: 10.1126/science.abf3363 33542131 PMC8097968

[107] Guthrie L Gupta S Daily J Kelly L . Human microbiome signatures of differential colorectal cancer drug metabolism. NPJ Biofilms Microbiomes. (2017) 3:27. doi: 10.1038/s41522-017-0034-1 29104759 PMC5665930

[108] Geller LT Barzily-Rokni M Danino T Jonas OH Shental N Nejman D . Potential role of intratumor bacteria in mediating tumor resistance to the chemotherapeutic drug gemcitabine. Science. (2017) 357:1156–60. doi: 10.1126/science.aah5043 28912244 PMC5727343

[109] Geller LT Straussman R . Intratumoral bacteria may elicit chemoresistance by metabolizing anticancer agents. Mol Cell Oncol. (2017) 5:e1405139. doi: 10.1080/23723556.2017.1405139 29404397 PMC5791857

[110] Spanogiannopoulos P Kyaw TS Guthrie BGH Bradley PH Lee JV Melamed J . Host and gut bacteria share metabolic pathways for anti-cancer drug metabolism. Nat Microbiol. (2022) 7:1605–20. doi: 10.1038/s41564-022-01226-5 36138165 PMC9530025

[111] Lehouritis P Cummins J Stanton M Murphy CT McCarthy FO Reid G . Local bacteria affect the efficacy of chemotherapeutic drugs. Sci Rep. (2015) 5:14554. doi: 10.1038/srep14554 26416623 PMC4586607

[112] Roichman A Zuo Q Hwang S Lu W Cordova RA MacArthur MR . Microbiome metabolism of dietary phytochemicals controls the anticancer activity of PI3K inhibitors. Cell. (2025) 188:3065–3080.e21. doi: 10.1016/j.cell.2025.04.041 40393457

[113] Li H He J Jia W . The influence of gut microbiota on drug metabolism and toxicity. Expert Opin Drug Metab Toxicol. (2016) 12:31–40. doi: 10.1517/17425255.2016.1121234 26569070 PMC5683181

[114] Stringer AM Gibson RJ Logan RM Bowen JM Yeoh AS Hamilton J . Gastrointestinal microflora and mucins may play a critical role in the development of 5-fluorouracil-induced gastrointestinal mucositis. Exp Biol Med (Maywood). (2009) 234:430–41. doi: 10.3181/0810-RM-301 19176868

[115] Maier L Pruteanu M Kuhn M Zeller G Telzerow A Anderson EE . Extensive impact of non-antibiotic drugs on human gut bacteria. Nature. (2018) 555:623–8. doi: 10.1038/nature25979 29555994 PMC6108420

[116] Montassier E Gastinne T Vangay P Al-Ghalith GA Bruley des Varannes S Massart S . Chemotherapy-driven dysbiosis in the intestinal microbiome. Aliment Pharmacol Ther. (2015) 42:515–28. doi: 10.1111/apt.13302 26147207

[117] Chang CW Lee HC Li LH Chiang Chiau JS Wang TE Chuang WH . Fecal microbiota transplantation prevents intestinal injury, upregulation of toll-like receptors, and 5-fluorouracil/oxaliplatin-induced toxicity in colorectal cancer. Int J Mol Sci. (2020) 21:386. doi: 10.3390/ijms21020386 31936237 PMC7013718

[118] Chenxiang D Jing Z Xiaojian Y Hua X Xi Y . Interplay between the abdominopelvic radiotherapy and gut microbiota. Front Microbiol. (2025) 16:1692179. doi: 10.3389/fmicb.2025.1692179 41459231 PMC12742314

[119] Wang L Wang X Zhang G Ma Y Zhang Q Li Z . The impact of pelvic radiotherapy on the gut microbiome and its role in radiation-induced diarrhoea: a systematic review. Radiat Oncol. (2021) 16:187. doi: 10.1186/s13014-021-01899-y 34563216 PMC8466721

[120] Wang Z Wang Q Wang X Zhu L Chen J Zhang B . Gut microbial dysbiosis is associated with development and progression of radiation enteritis during pelvic radiotherapy. J Cell Mol Med. (2019) 23:3747–56. doi: 10.1111/jcmm.14289 30908851 PMC6484301

[121] Ha-Young P Jin-Hee Y . X-ray radiation-induced intestinal barrier dysfunction in human epithelial Caco-2 cell monolayers. Ecotoxicology Environ Saf. (2023) 264:115404. doi: 10.1016/j.ecoenv.2023.115404 37625335

[122] Ma CY Zhao J Gan GH He XL Xu XT Qin SB . Establishment of a prediction model for severe acute radiation enteritis associated with cervical cancer radiotherapy. World J Gastroenterol. (2023) 29:1344–58. doi: 10.3748/wjg.v29.i8.1344 36925455 PMC10011961

[123] Eaton SE Kaczmarek J Mahmood D McDiarmid AM Norarfan AN Scott EG . Exploiting dietary fibre and the gut microbiota in pelvic radiotherapy patients. Br J Cancer. (2022) 127:2087–98. doi: 10.1038/s41416-022-01980-7 36175620 PMC9727022

[124] Li Y Zhang Y Wei K He J Ding N Hua J . Review: Effect of gut microbiota and its metabolite SCFAs on radiation-induced intestinal injury. Front Cell Infect Microbiol. (2021) 11:577236. doi: 10.3389/fcimb.2021.577236 34307184 PMC8300561

[125] Louis P Flint HJ . Formation of propionate and butyrate by the human colonic microbiota. Environ Microbiol. (2017) 19:29–41. doi: 10.1111/1462-2920.13589 27928878

[126] Parada Venegas D De la Fuente MK Landskron G González MJ Quera R Dijkstra G . Short chain fatty acids (SCFAs)-mediated gut epithelial and immune regulation and its relevance for inflammatory bowel diseases. Front Immunol. (2019) 10:277. doi: 10.3389/fimmu.2019.00277 30915065 PMC6421268

[127] Grass GD Krishna N Kim S . The immune mechanisms of abscopal effect in radiation therapy. Curr Probl Cancer. (2016) 40:10–24. doi: 10.1016/j.currproblcancer.2015.10.003 26612692

[128] Deng L Liang H Xu M Yang X Burnette B Arina A . STING-dependent cytosolic DNA sensing promotes radiation-induced type I interferon-dependent antitumor immunity in immunogenic tumors. Immunity. (2014) 41:843–52. doi: 10.1016/j.immuni.2014.10.019 25517616 PMC5155593

[129] Vanpouille-Box C Alard A Aryankalayil MJ Sarfraz Y Diamond JM Schneider RJ . DNA exonuclease Trex1 regulates radiotherapy-induced tumour immunogenicity. Nat Commun. (2017) 8:15618. doi: 10.1038/ncomms15618 28598415 PMC5472757

[130] Uribe-Herranz M Rafail S Beghi S Gil-de-Gómez L Verginadis I Bittinger K . Gut microbiota modulate dendritic cell antigen presentation and radiotherapy-induced antitumor immune response. J Clin Invest. (2020) 130:466–79. doi: 10.1172/JCI124332 31815742 PMC6934221

[131] Felchle H Gissibl J Lansink Rotgerink L Nefzger SM Walther CN Timnik VR . Influence of intestinal microbial metabolites on the abscopal effect after radiation therapy combined with immune checkpoint inhibitors. Clin Transl Radiat Oncol. (2024) 46:100758. doi: 10.1016/j.ctro.2024.100758 38500667 PMC10945164

[132] Zhao Y Chen F Wu W Sun M Bilotta AJ Yao S . GPR43 mediates microbiota metabolite SCFA regulation of antimicrobial peptide expression in intestinal epithelial cells via activation of mTOR and STAT3. Mucosal Immunol. (2018) 11:752–62. doi: 10.1038/mi.2017.118 29411774 PMC5976519

[133] Thangaraju M Cresci GA Liu K Ananth S Gnanaprakasam JP Browning DD . GPR109A is a G-protein-coupled receptor for the bacterial fermentation product butyrate and functions as a tumor suppressor in colon. Cancer Res. (2009) 69:2826–32. doi: 10.1158/0008-5472.CAN-08-4466 19276343 PMC3747834

[134] Abdeen SK Mastandrea I Stinchcombe N Puschhof J Elinav E . Diet-microbiome interactions in cancer. Cancer Cell. (2025) 43:680–707. doi: 10.1016/j.ccell.2025.03.013 40185096

[135] Papadimitriou N Markozannes G Kanellopoulou A Critselis E Alhardan S Karafousia V . An umbrella review of the evidence associating diet and cancer risk at 11 anatomical sites. Nat Commun. (2021) 12:4579. doi: 10.1038/s41467-021-24861-8 34321471 PMC8319326

[136] Dunneram Y Lee JY Watling CZ Lawson I Parsaeian M Fraser GE . Vegetarian diets and cancer risk: pooled analysis of 1.8 million women and men in nine prospective studies on three continents. Br J Cancer. (2026) 134(8):1218–29. doi: 10.1038/s41416-025-03327-4 41748939 PMC13035945

[137] Steck SE Murphy EA . Dietary patterns and cancer risk. Nat Rev Cancer. (2020) 20:125–38. doi: 10.1038/s41568-019-0227-4 31848467

[138] Yu Y Cai Y Yang B Xie S Shen W Wu Y . High-fat diet enhances the liver metastasis potential of colorectal cancer through microbiota dysbiosis. Cancers (Basel). (2022) 14:2573. doi: 10.3390/cancers14112573 35681554 PMC9179364

[139] Amar J Burcelin R Ruidavets JB Cani PD Fauvel J Alessi MC . Energy intake is associated with endotoxemia in apparently healthy men. Am J Clin Nutr. (2008) 87:1219–23. doi: 10.1093/ajcn/87.5.1219 18469242

[140] Jakobsdottir G Xu J Molin G Ahrné S Nyman M . High-fat diet reduces the formation of butyrate, but increases succinate, inflammation, liver fat and cholesterol in rats, while dietary fibre counteracts these effects. PloS One. (2013) 8:e80476. doi: 10.1371/journal.pone.0080476 24236183 PMC3827442

[141] Makki K Deehan EC Walter J Bäckhed F . The impact of dietary fiber on gut microbiota in host health and disease. Cell Host Microbe. (2018) 23:705–15. doi: 10.1016/j.chom.2018.05.012 29902436

[142] Ma W Nguyen LH Song M Wang DD Franzosa EA Cao Y . Dietary fiber intake, the gut microbiome, and chronic systemic inflammation in a cohort of adult men. Genome Med. (2021) 13:102. doi: 10.1186/s13073-021-00921-y 34140026 PMC8212460

[143] Turati F Galeone C Augustin LSA La Vecchia C . Glycemic index, glycemic load and cancer risk: an updated meta-analysis. Nutrients. (2019) 11:2342. doi: 10.3390/nu11102342 31581675 PMC6835610

[144] Singh RK Chang HW Yan D Lee KM Ucmak D Wong K . Influence of diet on the gut microbiome and implications for human health. J Transl Med. (2017) 15:73. doi: 10.1186/s12967-017-1175-y 28388917 PMC5385025

[145] Watson H Mitra S Croden FC Taylor M Wood HM Perry SL . A randomised trial of the effect of omega-3 polyunsaturated fatty acid supplements on the human intestinal microbiota. Gut. (2018) 67:1974–83. doi: 10.1136/gutjnl-2017-314968 28951525

[146] Tilg H Moschen AR . Food, immunity, and the microbiome. Gastroenterology. (2015) 148:1107–19. doi: 10.1053/j.gastro.2014.12.036 25575570

[147] Sun J Song S Liu J Chen F Li X Wu G . Gut microbiota as a new target for anticancer therapy: from mechanism to means of regulation. NPJ Biofilms Microbiomes. (2025) 11:43. doi: 10.1038/s41522-025-00678-x 40069181 PMC11897378

[148] Korem T Zeevi D Suez J Weinberger A Avnit-Sagi T Pompan-Lotan M . Growth dynamics of gut microbiota in health and disease inferred from single metagenomic samples. Science. (2015) 349:1101–6. doi: 10.1126/science.aac4812 26229116 PMC5087275

[149] Doel JJ Benjamin N Hector MP Rogers M Allaker RP . Evaluation of bacterial nitrate reduction in the human oral cavity. Eur J Oral Sci. (2005) 113:14–9. doi: 10.1111/j.1600-0722.2004.00184.x 15693824

[150] Su D Nie Y Zhu A Chen Z Wu P Zhang L . Vitamin D signaling through induction of Paneth cell defensins maintains gut microbiota and improves metabolic disorders and hepatic steatosis in animal models. Front Physiol. (2016) 7:498. doi: 10.3389/fphys.2016.00498 27895587 PMC5108805

[151] Gibson GR Roberfroid MB . Dietary modulation of the human colonic microbiota: introducing the concept of prebiotics. J Nutr. (1995) 125:1401–12. doi: 10.1093/jn/125.6.1401 7782892

[152] Gibson GR Hutkins R Sanders ME Prescott SL Reimer RA Salminen SJ . Expert consensus document: The International Scientific Association for Probiotics and Prebiotics (ISAPP) consensus statement on the definition and scope of prebiotics. Nat Rev Gastroenterol Hepatol. (2017) 14:491–502. doi: 10.1038/nrgastro.2017.75 28611480

[153] Sanders ME Merenstein DJ Reid G Gibson GR Rastall RA . Probiotics and prebiotics in intestinal health and disease: from biology to the clinic. Nat Rev Gastroenterol Hepatol. (2019) 16:605–16. doi: 10.1038/s41575-019-0173-3 31296969

[154] Bedu-Ferrari C Biscarrat P Langella P Cherbuy C . Prebiotics and the human gut microbiota: From breakdown mechanisms to the impact on metabolic health. Nutrients. (2022) 14:2096. doi: 10.3390/nu14102096 35631237 PMC9147914

[155] Donohoe DR Garge N Zhang X Sun W O'Connell TM Bunger MK . The microbiome and butyrate regulate energy metabolism and autophagy in the mammalian colon. Cell Metab. (2011) 13:517–26. doi: 10.1016/j.cmet.2011.02.018 21531334 PMC3099420

[156] Cherbuy C Honvo-Houeto E Bruneau A Bridonneau C Mayeur C Duée PH . Microbiota matures colonic epithelium through a coordinated induction of cell cycle-related proteins in gnotobiotic rat. Am J Physiol Gastrointest Liver Physiol. (2010) 299:G348–57. doi: 10.1152/ajpgi.00384.2009 20466941

[157] Tegegne BA Abebaw D Teffera ZH Fenta A Belew H Belayneh M . Microbial therapeutics in cancer: Translating probiotics, prebiotics, synbiotics, and postbiotics from mechanistic insights to clinical applications: A topical review. FASEB J. (2025) 39:e71146. doi: 10.1096/fj.202502118R 41134211 PMC12551701

[158] Zhang Y Xu X Wang S Yin X Zhang B Zhu Z . Fecal microbiota transplantation combined with anti-PD-1 therapy in refractory microsatellite-stable gastric cancer: a phase I feasibility and safety study. J Immunother Cancer. (2026) 14:e013823. doi: 10.1136/jitc-2025-013823 41871875 PMC13034380

[159] Porcari S Ciccarese C Heidrich V Rondinella D Quaranta G Severino A . Fecal microbiota transplantation plus pembrolizumab and axitinib in metastatic renal cell carcinoma: the randomized phase 2 TACITO trial. Nat Med. (2026) 32(4):1316–24. doi: 10.1038/s41591-025-04189-2 41606119 PMC13099650

[160] Duttagupta S Messaoudene M Hunter S Desilets A Jamal R Mihalcioiu C . Fecal microbiota transplantation plus immunotherapy in non-small cell lung cancer and melanoma: the phase 2 FMT-LUMINate trial. Nat Med. (2026) 32(4):1337–50. doi: 10.1038/s41591-025-04186-5 41606121 PMC13099432

[161] Ianiro G Rossi E Thomas AM Schinzari G Masucci L Quaranta G . Faecal microbiota transplantation for the treatment of diarrhoea induced by tyrosine-kinase inhibitors in patients with metastatic renal cell carcinoma. Nat Commun. (2020) 11:4333. doi: 10.1038/s41467-020-18127-y 32859933 PMC7455693

[162] Reddi S Senyshyn L Ebadi M Podlesny D Minot SS Gooley T . Fecal microbiota transplantation to prevent acute graft-versus-host disease: pre-planned interim analysis of donor effect. Nat Commun. (2025) 16:1034. doi: 10.1038/s41467-025-56375-y 39863610 PMC11762788

[163] Rashidi A Ebadi M Rehman TU Elhusseini H Kazadi D Halaweish H . Randomized double-blind phase II trial of fecal microbiota transplantation versus placebo in allogeneic hematopoietic cell transplantation and AML. J Clin Oncol. (2023) 41:5306–19. doi: 10.1200/JCO.22.02366 37235836 PMC10691796

[164] Routy B Lenehan JG Miller WH Jr Jamal R Messaoudene M . Fecal microbiota transplantation plus anti-PD-1 immunotherapy in advanced melanoma: a phase I trial. Nat Med. (2023) 29:2121–32. doi: 10.1038/s41591-023-02453-x 37414899

[165] Malard F Vekhoff A Lapusan S Isnard F D'incan-Corda E Rey J . Gut microbiota diversity after autologous fecal microbiota transfer in acute myeloid leukemia patients. Nat Commun. (2021) 12:3084. doi: 10.1038/s41467-021-23376-6 34035290 PMC8149453

[166] Fernandes R Jabbarizadeh B Rajeh A Hong MMY Baines KJ Ernst S . Fecal microbiota transplantation plus immunotherapy in metastatic renal cell carcinoma: the phase 1 PERFORM trial. Nat Med. (2026) 32(4):1325–36. doi: 10.1038/s41591-025-04183-8 41606120 PMC13099417

[167] Zhao W Lei J Ke S Chen Y Xiao J Tang Z . Fecal microbiota transplantation plus tislelizumab and fruquintinib in refractory microsatellite stable metastatic colorectal cancer: an open-label, single-arm, phase II trial (RENMIN-215). EClinicalMedicine. (2023) 66:102315. doi: 10.1016/j.eclinm.2023.102315 38024475 PMC10679864

[168] Yang B Jang MH Kim SJ Choi Y Hwang J Kim A . 593 First-in-human evaluation of the safety, tolerability, and gut microbial composition of a live biotherapeutic product GB104 in patients with colorectal cancer. J Immunother Cancer. (2024) 12. doi: 10.1136/jitc-2024-SITC2024.0593

[169] Lee JB Baek S Kim DK Kwon BE Ahn JS Nagasaka M . Phase I trial of CJRB-101 plus pembrolizumab in patients with metastatic non-small cell lung cancer, head and neck squamous cell carcinoma and melanoma. J Immunother Cancer. (2026) 14:e014702. doi: 10.1136/jitc-2025-014702 42140743 PMC13182499

[170] Lyon De Ana C Prince A Darrah E Buresh S Menon R Norman J . 607 CONSORTIUM-IO: a phase 1 study evaluating a combination of an 11-strain bacterial consortium (VE800) and nivolumab in treatment of select refractory or metastatic cancers. J Immunother Cancer. (2024) 12. doi: 10.1136/jitc-2024-SITC2024.0607

[171] Ebrahimi H Dizman N Meza L Malhotra J Li X Dorff T . Cabozantinib and nivolumab with or without live bacterial supplementation in metastatic renal cell carcinoma: a randomized phase 1 trial. Nat Med. (2024) 30:2576–85. doi: 10.1038/s41591-024-03086-4 38942995 PMC11405272

[172] Dizman N Meza L Bergerot P Alcantara M Dorff T Lyou Y . Nivolumab plus ipilimumab with or without live bacterial supplementation in metastatic renal cell carcinoma: a randomized phase 1 trial. Nat Med. (2022) 28:704–12. doi: 10.1038/s41591-022-01694-6 35228755 PMC9018425

[173] Xia C Jiang C Li W Wei J Hong H Li J . A phase II randomized clinical trial and mechanistic studies using improved probiotics to prevent oral mucositis induced by concurrent radiotherapy and chemotherapy in nasopharyngeal carcinoma. Front Immunol. (2021) 12:618150. doi: 10.3389/fimmu.2021.618150 33841399 PMC8024544

[174] Mego M Kasperova B Chovanec J Danis R Reckova M Bystricky B . The beneficial effect of probiotics in the prevention of irinotecan-induced diarrhea in colorectal cancer patients with colostomy: a pooled analysis of two probiotic trials (Probio-SK-003 and Probio-SK-005) led by Slovak Cooperative Oncology Group. Front Oncol. (2024) 14:1438657. doi: 10.3389/fonc.2024.1438657 39104721 PMC11298351

[175] Yang Y Xia Y Chen H Hong L Feng J Yang J . The effect of perioperative probiotics treatment for colorectal cancer: short-term outcomes of a randomized controlled trial. Oncotarget. (2016) 7:8432–40. doi: 10.18632/oncotarget.7045 26824990 PMC4885004

[176] Gianotti L Morelli L Galbiati F Rocchetti S Coppola S Beneduce A . A randomized double-blind trial on perioperative administration of probiotics in colorectal cancer patients. World J Gastroenterol. (2010) 16:167–75. doi: 10.3748/wjg.v16.i2.167 20066735 PMC2806554

[177] Pereira Riveros T Jané Salas E Lozano Borbalas A Aguilera FR Vinuesa Aumedes T . Effect of a probiotic combination on clinical and microbiological oral parameters in head and neck cancer patients: a randomised clinical trial. Cancers (Basel). (2025) 17:2459. doi: 10.3390/cancers17152459 40805159 PMC12346730

[178] Goto Y Dolton G Thomas H Morin T Tajima Y Imamura K . A probiotic bacterium modulates antitumour γδ T-cell responses in lung cancer. Front Immunol. (2026) 17:1750569. doi: 10.3389/fimmu.2026.1750569 41993168 PMC13079699

[179] Osterlund P Ruotsalainen T Korpela R Saxelin M Ollus A Valta P . Lactobacillus supplementation for diarrhoea related to chemotherapy of colorectal cancer: a randomised study. Br J Cancer. (2007) 97:1028–34. doi: 10.1038/sj.bjc.6603990 17895895 PMC2360429

[180] Cervantes-Barragan L Chai JN Tianero MD Di Luccia B Ahern PP Merriman J . Lactobacillus reuteri induces gut intraepithelial CD4+CD8αα+ T cells. Science. (2017) 357:806–10. doi: 10.1126/science.aah5825 28775213 PMC5687812

[181] Elkrief A Pidgeon R Maleki Vareki S Messaoudene M Castagner B Routy B . The gut microbiome as a target in cancer immunotherapy: opportunities and challenges for drug development. Nat Rev Drug Discov. (2025) 24:685–704. doi: 10.1038/s41573-025-01211-7 40457025

[182] Duvallet C Zellmer C Panchal P Budree S Osman M Alm EJ . Framework for rational donor selection in fecal microbiota transplant clinical trials. PloS One. (2019) 14:e0222881. doi: 10.1371/journal.pone.0222881 31600222 PMC6786724

[183] Cordaillat-Simmons M Rouanet A Pot B . Live biotherapeutic products: the importance of a defined regulatory framework. Exp Mol Med. (2020) 52:1397–406. doi: 10.1038/s12276-020-0437-6 32908212 PMC8080583

[184] Routy B Le Chatelier E Derosa L Duong CPM Alou MT Daillère R . Gut microbiome influences efficacy of PD-1-based immunotherapy against epithelial tumors. Science. (2018) 359:91–7. doi: 10.1126/science.aan3706 29097494

[185] Spreafico A Heirali AA Araujo DV Tan TJ Oliva M Schneeberger PHH . First-in-class Microbial Ecosystem Therapeutic 4 (MET4) in combination with immune checkpoint inhibitors in patients with advanced solid tumors (MET4-IO trial). Ann Oncol. (2023) 34:520–30. doi: 10.1016/j.annonc.2023.02.011 36863483

[186] Freedman SB Williamson-Urquhart S Farion KJ Gouin S Willan AR Poonai N . Multicenter trial of a combination probiotic for children with gastroenteritis. N Engl J Med. (2018) 379:2015–26. doi: 10.1056/NEJMoa1802597 30462939

[187] Spencer CN McQuade JL Gopalakrishnan V McCulloch JA Vetizou M Cogdill AP . Dietary fiber and probiotics influence the gut microbiome and melanoma immunotherapy response. Science. (2021) 374:1632–40. doi: 10.1126/science.aaz7015 34941392 PMC8970537

[188] Kapoor A Gupta A Sansar B Mishra BK Kallapura G Giridhar P . Translational implications of microbiome testing methodologies in lung cancer: a systematic review of 16S rRNA sequencing, shotgun metagenomics, and short-chain fatty acid profiling. JCO Oncol Adv. (2026). 3(1):e2500194. doi: 10.1200/OA-25-00194

[189] Greathouse KL Choudhury A . Precision nutrition and the gut microbiome: Harnessing AI to revolutionize cancer prevention and therapy. Cell Host Microbe. (2025) 33:766–76. doi: 10.1016/j.chom.2025.05.011 40505617

[190] Huang L Li Y Zhang C Jiang A Zhu L Mou W . Microbiome meets immunotherapy: unlocking the hidden predictors of immune checkpoint inhibitors. NPJ Biofilms Microbiomes. (2025) 11:180. doi: 10.1038/s41522-025-00819-2 40897718 PMC12405452

[191] Wang X He J Ding G Tang Y Wang Q . Artificial intelligence-enabled multi-omics for predicting immune checkpoint inhibitor response and resistance. J Multidiscip Healthc. (2026) 19:572089. doi: 10.2147/JMDH.S572089 41777263 PMC12951862

[192] Gunjur A Shao Y Rozday T Klein O Mu A Haak BW . A gut microbial signature for combination immune checkpoint blockade across cancer types. Nat Med. (2024) 30:797–809. doi: 10.1038/s41591-024-02823-z 38429524 PMC10957475

[193] Litchfield K Reading JL Puttick C Thakkar K Abbosh C Bentham R . Meta-analysis of tumor- and T cell-intrinsic mechanisms of sensitization to checkpoint inhibition. Cell. (2021) 184:596–614.e14. doi: 10.1016/j.cell.2021.01.002 33508232 PMC7933824

[194] Cao S Liu J Li Y . Harnessing multi-omics and machine learning for predicting immune checkpoint blockade responses: advances, challenges, and future directions. Fundam Res. (2025) 6:62–76. doi: 10.1016/j.fmre.2025.08.009 41647536 PMC12869780

[195] Sheller MJ Edwards B Reina GA Martin J Pati S Kotrotsou A . Federated learning in medicine: facilitating multi-institutional collaborations without sharing patient data. Sci Rep. (2020) 10:12598. doi: 10.1038/s41598-020-69250-1 32724046 PMC7387485

[196] Bolyen E Rideout JR Dillon MR Bokulich NA Abnet CC Al-Ghalith GA . Reproducible, interactive, scalable and extensible microbiome data science using QIIME 2. Nat Biotechnol. (2019) 37:852–7. doi: 10.1038/s41587-019-0209-9 31341288 PMC7015180

[197] Limeta A Gatto F Herrgård MJ Ji B Nielsen J . Leveraging high-resolution omics data for predicting responses and adverse events to immune checkpoint inhibitors. Comput Struct Biotechnol J. (2023) 21:3912–9. doi: 10.1016/j.csbj.2023.07.032 37602228 PMC10432706

[198] Zhou T Zhao F . AI-empowered human microbiome research. Gut. (2025) 75(7):1432–46. doi: 10.1136/gutjnl-2025-335946 40983504 PMC13311949

[199] Cheng F Soleimani Samarkhazan H Khazaei Y . CRISPR-engineered microbiome: living therapeutics revolutionize blood cancer immunotherapy. NPJ Biofilms Microbiomes. (2025) 12:17. doi: 10.1038/s41522-025-00882-9 41387457 PMC12816696

